# Explainable deep learning framework incorporating medical knowledge for insulin titration in diabetes

**DOI:** 10.1038/s43856-026-01449-1

**Published:** 2026-02-26

**Authors:** Haowei He, Zhen Ying, Biao Li, Yujuan Fan, Ping Wang, Jiaping Lu, Liming Wu, Hexu Zhao, Yanying Guo, Guangyu Wang, Yang Yuan, Ying Chen, Xiaoying Li

**Affiliations:** 1 IIIS, Tsinghua University, Beijing, China; 2 Ministry of Education Key Laboratory of Metabolism and Molecular Medicine, Department of Endocrinology and Metabolism, Zhongshan Hospital, Fudan University, Shanghai, China; 3 Shanghai Qi Zhi Institute, Shanghai, China; 4 Department of Endocrinology and Metabolism, Minhang Hospital, Fudan University, Shanghai, China; 5 Department of Endocrinology and Metabolism, Qingpu Branch of Zhongshan Hospital Affiliated to Fudan University, Shanghai, China; 6 Department of Endocrinology and Metabolism, Shanghai Xuhui Central Hospital, Shanghai, China; 7 Department of Endocrinology and Metabolic Diseases, People’s Hospital of Xinjiang Uygur Autonomous Region, Xinjiang Clinical Research Center for Diabetes, Urumqi, China; 8 State Key Laboratory of Networking and Switching Technology, Beijing University of Posts and Telecommunications, Beijing, China; 9 Shanghai Key Laboratory of Metabolic Remodeling and Health, Institute of Metabolism and Integrative Biology, Fudan University, Shanghai, China

**Keywords:** Type 2 diabetes, Therapeutics

## Abstract

**Background:**

Deep learning has shown promise in diabetes management but faces challenges in real-world application due to its “black-box” nature, characterized by opaque internal decision-making processes. Explainable artificial intelligence (XAI) methods have been proposed to enhance model transparency. However, most of current XAI methods applied in the medical field often ignore the interaction of features in complex environments and pose deviation from clinical domain knowledge.

**Methods:**

Our study used two Electronic Health Record (EHR) cohorts of hospitalized patients with type 2 diabetes (T2DM), including an internal dataset of 1,275 inpatients (mean age 58.5 ± 14.3 years) and an external dataset of 292 patients (mean age 69.3 ± 14.5 years). We introduce an expert-guided XAI framework to improve the transparency and trustworthiness of deep learning models for insulin titration in diabetes management. The framework utilizes a post-hoc XAI model named Shapley Taylor Interaction Index (STII) to capture the impact of feature interactions. Additionally, the model is refined iteratively in a doctor-in-the-loop (DIL) process by encoding clinical constraints to align with medical expertise.

**Results:**

Here we show that our STII-DIL model could explore the interaction factors and reduce unreasonable explanations compared with other explanation models. The final XAI system explanations demonstrated strong alignment with experts’ explanations and increased correctness by expert evaluation An AI-human collaboration study revealed that insulin titration accuracy significantly improved for junior clinicians with STII-DIL assistance, while senior clinicians showed minimal change. Both junior and senior clinicians reported increased confidence when using the STII-DIL system.

**Conclusions:**

We present an explainable deep learning framework that combines post-hoc XAI and expert domain knowledge to provide transparent and expert-aligned explanations for insulin titration in type 2 diabetes management. This framework enhances decision-making accuracy and confidence, especially for junior clinicians, and may facilitate broader clinical adoption of AI-assisted decision-making tools.

## Abstract (web summary)

He, Ying et al. develop an explainable deep learning framework that combines post-hoc explainable AI and expert domain knowledge for insulin titration in type 2 diabetes management. This framework improves clinical decision-making, especially for junior clinicians, and may facilitate broader clinical adoption of AI-assisted decision-making tools.

## Plain language summary

This study addresses the need for transparent and reliable artificial intelligence (AI) tools in diabetes care. We developed an explainable deep learning system that helps doctors adjust insulin doses by showing how different clinical factors contribute to its recommendations. The system combines an AI explanation method with guidance from medical experts, allowing the model to be refined to better match real clinical reasoning. We found that this approach produced clearer and more accurate explanations and supported better decision-making, especially for junior clinicians. Both junior and senior clinicians also reported greater confidence when using the system. These findings suggest that explainable AI may improve safety and support wider clinical use of AI-assisted diabetes management.

## Introduction

Type 2 diabetes (T2D) is a highly prevalent chronic disease that significantly impacts global mortality and societal burden^1^. Optimized insulin treatment is crucial for improved glycemic control and thus prevents associated comorbidities and mortality in T2D patients. Traditional insulin treatment for T2D patients typically relies on clinical guidance combined with physicians’ experience, which remains challenging and time-consuming for limited medical resource to adjust optimized insulin dosages^2,3^. Artificial intelligence (AI) approaches have emerged to offer promising powerful tools to aid in disease diagnosis and management. Recent applications include diabetic retinopathy prediction in type 1 diabetes using CGM-based machine learning approaches and combining genetic and glucose profiling to enhance immunological risk prediction^4,5^.

We recently developed a deep-learning-based system for personalized and dynamic insulin dosing for T2D patients and it has demonstrated remarkable performance in optimizing glycemic control^6^. Yet the black-box nature of deep learning poses challenges on explainability and broader clinical integration^7,8^. It is generally considered that a “black box” model could lead clinicians’ mistrust and hesitation when reviewing the AI decisions^9^. Specifically, the limitations of “black-box” models can be quantitatively characterized by their lack of transparency, limited alignment with expert reasoning, and reduced interpretability—factors that can be benchmarked through computational fidelity metrics and human-centered evaluations of clinicians’ understanding, trust, and decision confidence^10^. Thus, the European Union’s General Data Protection Regulation (GDPR) law requests an interpretation of algorithm based decision-making process before patient care^11^. Explainable AI (XAI) methods have been proposed to make the reasoning of deep learning system more transparent, which allow the deep-learning-based decisions understandable by clinicians and thus can strengthen their trust in the system^12^.

Several interpretability algorithms have been developed to address the “black box” challenge^13,14^. Recent algorithms can be further classified into inherently interpretable (Ante-hoc) and post-hoc methods^15^. Ante-hoc methods refer to models whose structures are relatively simple and easy to understand, including classical decision trees, linear regression, Bayesian models, and so on^13,16,17^ While these models provide architectural transparency, their simplicity inherent may lead to underperformance in the face of complex problems^18,19^. To address the inherent trade-off between model performance and interpretability, post-hoc methods have been developed to elucidate the decision-making processes of complex models after their training is completed. These methods could aid in understanding models that are inherently intricate and challenging to comprehend directly. For example, integrated gradients (IG) quantify feature importance through input-to-baseline gradient integration^12^, while shapley additive explanations (SHAP) allocates predictive contributions using game-theoretic principles to ensure equitable feature importance distribution^15^. Although these interpretation algorithms have proven valuable in some medical tasks^19–23^, the majority of them focus on diagnosis and prediction, with limited application in supporting treatment decisions. Moreover, current post-hoc interpretation algorithms encounter two critical challenges in clinical practice. Firstly, complex diseases typically involve the interplay of multiple factors, but most algorithms analyze only single factors, risking oversimplified or incomplete explanations. Secondly, these algorithms depend entirely on computational approaches, which may produce interpretations inconsistent with established medical knowledge. This discrepancy can lead to significant confusion and potentially misleading conclusions, particularly when directly deployed in clinical settings. Thus, there is an urgent need for novel approaches that can enhance XAI interpretations to offer comprehensive and accurate explanations for practical clinical use.

In this study, we introduce a doctor-in-the-loop (DIL) XAI framework to address these challenges and adapt the explanation model in insulin titration. First, we utilize a second-order explainable algorithm named Shapley Taylor Interaction Index (STII) as the base algorithm to comprehensively present the effects of single and interaction factors. Compared with other second-order or attention-based interpretability approaches, STII achieves a more balanced compromise between theoretical rigor and computational feasibility, offering mathematically grounded and quantitatively consistent attributions that are particularly suited for clinical interpretation^24–27^. Then, we incorporate expert-guided domain knowledge to refine the explainable algorithm. Furthermore, we conduct the statistical analysis, expert evaluation and AI-human collaboration study to demonstrate the accuracy and feasibility of our explanation model. In summary, the final STII-DIL explanations demonstrates strong alignment with experts’ explanations and are deemed increased correctness by expert evaluation. We also conduct an AI-human collaboration study to investigate the influence of our STII-DIL explainable system on junior and senior physicians in clinical use, and find that the decision accuracy and confidence significantly improve in junior physicians with STII-DIL assistance. Our study is an attempt to incorporated expert-guided domain knowledge to adapt post-hoc XAI algorithm, aiming to develop a well-performed XAI support system aiding in deep learning based clinical decision making.

## Methods

### Overall study design

**Fig. 1:**
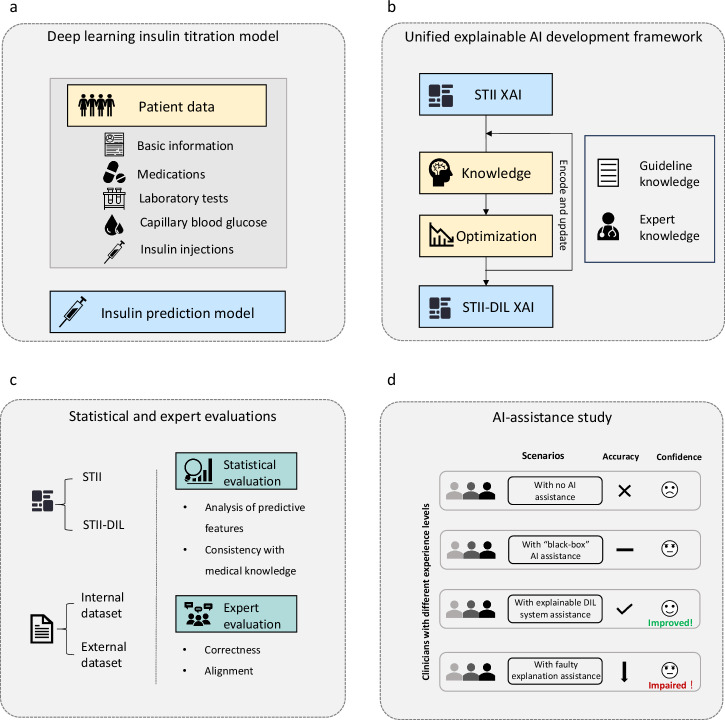
Overall study design. Our research encompassed a four-stage methodology. **a** EHR datasets of hospitalized patients with T2DM were used to predict insulin dosage based on a previous deep learning model. **b** Our explainability pipeline, based on the second-order STII framework, encoded medical knowledge from guidelines and expert insights to optimize its performance. **c** We conducted statistical analyses and expert evaluations comparing the original and final versions of the explanation to demonstrate the feasibility of the pipeline. **d** We further conducted an AI-assistance study to evaluate the utility of our explainable STII-DIL system in clinical use.

Our study consisted of four parts as shown in Fig. 1. First, we collected EHR datasets of hospitalized patients with T2DM and developed the “black box” insulin prediction model^6^. Second, we created an XAI framework based on the second-order STII algorithm and encoded clinical knowledge to provide optimal explanations of our deep-learning-based model Third, we conducted statistical evaluations and expert evaluations to assess the performance of our explainable algorithm in the internal and external retrospective datasets. Finally, we conducted an AI-assistance study to evaluate the practical application value of our explainable algorithm in clinical use.

### Data sources

Our study included two EHR datasets of hospitalized patients with T2DM. The internal dataset comprised 1275 T2D inpatients from Zhongshan hospital, with a mean age of 58.5 ± 14.3 years, of which 41.1% were female. For the external dataset, 292 T2D patients from Xuhui Hospital and Qingpu Hospital were included to assess the generalizability of the model, featuring a mean age of 69.3 ± 14.5 years with 50.7% being female. The detail demographics and clinical characteristics of patients are presented in Table 1. The retrospective data and study design received IRB approval from Zhongshan Hospital, Shanghai, China (2019-014 R, 2024-118 R); XuHui Central Hospital, Shanghai, China (2021-007); and Qingpu Branch of Zhongshan Hospital, Shanghai, China (2021-25). Patient informed consent was waived by the Ethics Committees of Zhongshan Hospital, XuHui Central Hospital and Qingpu Branch of Zhongshan Hospital, due to the retrospective nature of the study and the inability to trace individual data.

**Table 1:** Dataset characteristics

	Internal validation set	External validation set	P value
Participants	1275	292	N/A
Insulin counts	19,591	5088	N/A
Age (year)	58.5 (14.3)	69.3 (14.5)	<0.001
Sex			<0.001
Male (%)	747 (58.6%)	144 (49.3%)	
Female (%)	528 (41.4%)	148 (50.7%)	
Weight (kg)	69.0 (15.4)	66.4 (15.0)	0.044
BMI (kg/m^2^)	25.5 (5.3)	23.8 (4.4)	<0.001
HbA1c (%)	9.3 (2.2)	9.2 (2.4)	0.781

Data are n (%) or mean (SD). P values were calculated using two-sided t-tests, or chi-square tests as appropriate. *BMI* body mass index, *HbA1c* glycated hemoglobin, *N/A* not applicable.

### Explanation development methods

The overview of explanation development process is presented in Supplementary Fig. 1.

#### Shapley Taylor Interaction Index (STII)

We utilized the Shapley Taylor Interaction Index (STII)^28^ to quantify the contribution of each input feature to the model’s predictions. Unlike traditional Shapley values that assess individual feature contributions, STII evaluates both individual and interactive effects, providing a more nuanced explanation suitable for complex models like insulin dose prediction (Supplementary Fig. 1b). Given an input $x$, we denote the whole feature set as $N$. Then the STII value for a subset of features $S$ with respect to $N$ for a deep network $f$ is defined as:

$$I\left(f,\,S\right):={\sum }_{T\in N{{{\rm{\backslash }}}}S}\frac{\left(n-t-s\right)!t!}{\left(n-s+1\right)!}{\delta }_{S}f\left(T\right)$$

where the lowercase letters represent the cardinalities of the sets, and ${\delta }_{S}f(T)$ is the discrete derivative for set $T$ with respect to set $S$. Simply put, the equation calculates the average marginal contribution of a feature set $S$ across all possible feature combinations. In our case, $f$ refers to the “black box” insulin prediction model, $S$ and $T$ are feature sets of a patient. The STII value $I\left(f,{S}\right)$ is calculated for each injection of each patient. More details can be found in article^28^.

#### Kernel and resampling acceleration algorithm

Given the exponential computation demands of calculating Shapley values, we implemented a kernel and resampling acceleration algorithm^24,28^. This approach uses weighted regression, optimized through LASSO regression, to efficiently estimate the contributions of feature combinations $I\left(f,{S}\right)$ (Supplementary Fig. 1c).

#### Integration of Expert Knowledge

Recognizing the limitations of traditional game theory-based models in healthcare, we incorporated medical knowledge into the STII calculations. This was achieved by embedding knowledge from guidelines and experts as constraints in the LASSO regression, ensuring that the generated explanations adhere to clinical understanding and common medical practice (Supplementary Fig. 1d).

The optimization problem for our algorithm was formulated as follows:

$$\min L\left(\alpha \right) =	{\sum }_{m=1}^{M}\frac{d-1}{\left(\begin{array}{c}d\\ {\left|\left|{x}_{m}\right|\right|}_{0}\end{array}\right){\left|\left|{x}_{m}\right|\right|}_{0}(d-{\left|\left|{x}_{m}\right|\right|}_{0})}( < \alpha ,\,{x}_{m} > -{y}_{m}) \\ 	+{\sum }_{k=1}^{K}{\lambda }_{k}{Constrai}{{nt}}_{k}(\alpha )$$

where $\alpha \in {R}^{{d}^{2}+d}$ represents the explanation coefficients for both single and interaction factors, $({x}_{m},\,{y}_{m})$ are $M$ resampled samples with some substituted dimensions of input $x$, ${\left|\left|\cdot \right|\right|}_{0}$ represents the number features that have not been substituted, and ${\lambda }_{k}$ controls the constraint strength. The detailed process of constraint generation is in the following.

#### Doctor in the Loop (DIL) Process

The DIL process played a critical role in optimizing the algorithm. During the iteration of the algorithm, a panel of three board-certified experts evaluated the explanations generated by our model based on evidence-based clinical guidelines and consensus statements. Experts assessed each explanation in terms of feature name, effect direction, and effect magnitude, and provided structured feedback through a standardized questionnaire and scoring rubric (see Supplementary Note 1). Expert feedback was used to iteratively update the algorithm, ensuring that the model’s outputs aligned with medical common sense and evidence-based practice. Through structured discussions, the experts and engineers refined and optimized constraints, which were derived from medical knowledge and encoded into the model. The process continued until the expert panel confirmed that the explanations were aligned with medical common sense. Its core purpose was to integrate well-established medical constraints into the algorithm, rather than relying on subjective experience, thereby enhancing the model’s reliability and clinical applicability. The final set of constraints was derived from established clinical knowledge and evidence-based guidelines^29–31^, ensuring their generalizability across different hospitals, healthcare systems, and patient populations. The detailed statements of ${Constraint}(\alpha )$ are in Supplementary Data 3.

### Comparison with other algorithms

To compare the performance of our method with other interpretability algorithms, we selected two widely used approaches: IG and SHAP. IG is an algorithm that attributes the contribution of input features to a neural network’s output by calculating and integrating the gradients of the model’s predictions from a baseline input to the actual input. This method is particularly effective for deep learning models as it provides a more focused explanation of the impact of each feature on the prediction and is favored for its ability to handle high-dimensional data^32^. SHAP is an algorithm based on cooperative game theory, which aims to explain the contribution of individual features to the model’s predictions by assigning each feature a Shapley value. One of the key advantages of SHAP is its ability to provide consistent and locally accurate explanations, making it a popular choice for explaining complex models, such as deep neural networks and ensemble methods^33^. We compared the contribution values of predictive features determined by IG, SHAP, and our method to assess the performance of these explainable algorithms. Our analysis included a comparison of the top 20 important features for each algorithm on population ranking charts. We also conducted permutation importance based on our neural network model to further examine feature relevance. Additionally, we employed Pearson correlation analysis for individual factors to evaluate the rationality of each algorithm’s explanation for single factors. We further employed dot plots and analyzed a patient case to demonstrate our algorithm’s capacity in elucidating feature interactions. We also replaced the LASSO algorithm (which is commonly used in Shapley value calculation) with the random forest algorithm to further validate robustness.

### Loop upgrade evaluation

To demonstrate the progressive improvement of the explainable algorithm’s understanding of domain knowledge in the loop, we compared the original and final versions of the explainable algorithm from two perspectives.

#### Statistical evaluations

We performed statistical evaluations on all patients in both the internal and external validation datasets to assess the impact of integrating various constraints into the algorithm. These constraints included changes in blood glucose (BG) levels, adjustments in antidiabetic medications, accounting for missing content, analysis of prescribed BG records, and examining the BG-insulin interaction. Specifically, we determined the impact of BG changes/antidiabetic medication constraints by comparing the distributions of BG/medication points between the original and final versions of the DIL algorithm. To assess the impact of missing content/prescribed BG constraints, we compared the proportions of missing items/prescribed BG records in both versions of the algorithm. Furthermore, the interaction between BG and insulin was elucidated through a detailed analysis of representative cases from both the original and final versions of the algorithm.

#### Expert evaluations

Three endocrinologists, each with 8, 10, and 21 years of clinical experience respectively, formed the evaluation panel for the specialist assessment. To explore the effect of adding constraints, we sampled 20 cases each from the internal validation set and the external validation set and compared the quality of explanations between the original and final versions. The expert panel was asked to assess the alignment and correctness of each case across the original and final versions of explanations. To minimize evaluation bias, the order of different versions of the explanation was disturbed and specialists were blinded to the version of the explanation. The detailed definitions of evaluation metrics are as follows:

1. *Alignment rate*: To evaluate the explanations’ alignment with medical knowledge, we used questionnaire 2 (item 1), which asked the reviewers to pick the top 3 influential features for each insulin prediction record based on the medical guidance and physicians’ expertise. The alignment rate is assessed by the degree of overlap between experts’ opinions and the top N algorithm-generated items. Specially, top N alignment rate (%) = the number of overlap items between experts and algorithm / the total number of influential items made by expert *100% (N = 3,5,10).

2. *Correctness rate*: To evaluate the explanations’ correctness, we used questionnaire 2 (item 2), which asked the reviewers whether the explanation items generated by the algorithm were correct from three aspects (feature, effect direction, and effect size), based on the medical guidance and physicians’ expertise. Correctness is determined by majority vote. The correctness rate was calculated as a percentage of correct items to all items, grouped into the following categories: (1) partial correctness rate: percentage of items with features deemed to affect insulin dosage, (2) moderate correctness: percentage of items with features deemed to affect insulin dosage and appropriate directions of effects (3) absolute correctness: percentage of items with features deemed to affect insulin dosage, appropriate directions of effects, as well as suitable effect sizes. Specially, correctness rate (%) = the number of correct items / the total number of items *100%.

See Supplementary Note 2 for detailed questionnaires.

### Clinical evaluation

#### Model deployment

The explainable insulin titration model was deployed in Zhongshan Hospital in December 2022. This AI system utilizes real-time patient information as input and provides insulin dosage regimens and explanations as output. Clinicians can interact with this AI system through the interface (Fig. 5). Figure 5a showcases the patient’s information collected upon admission, including demographic details, biochemical tests, capillary BG records, as well as insulin and medication usage records from the past three days. By clicking on the insulin recommendation window, clinicians can access the recommended insulin dose generated by the predictive modeling. For a more comprehensive understanding, clinicians can click on the “Expand explanation” button, enabling them to review the ten most critical explanation items generated by the current version of the explainable model. These items are derived from the patient’s information and dosage predictions provided by the predictive model (Fig. 5b). This interactive interface enhances the transparency and accessibility of the AI-generated recommendations, supporting clinicians in making informed decisions regarding insulin dosage based on a holistic explanation of patient data.

#### AI-assistance study

A multi-reader multi-case AI-assistance study was conducted to investigate the clinical significance of the final version of the explainable algorithm. 40 cases comprising 1,030 explanation items were included in this evaluation. An expert consensus panel of three endocrinology specialists provided their own recommended insulin dosage. This was used as a reference insulin dosage to assess the accuracy of clinician dosage. Eight clinicians were enrolled and divided into two groups: a junior group consisting of four less-experienced clinicians with 1-3 years of clinical experience, and a senior group consisting of four experienced clinicians with 4-6 years of clinical experience.

In the study, our AI system could provide insulin dosage recommendations and related explanation items. Clinicians’ decision process was divided into three steps. First, clinicians provided insulin dosage recommendations and rated their confidence directly based on the patients’ information with no AI assistance (scenario 1). Second, after viewing the sole insulin dosage recommendation by the AI system, clinicians gave their insulin dosage and confidence again as the results with plain AI dosage assistance (scenario 2). Third, clinicians viewed both the insulin dosage recommendation and related explanation items and gave their insulin dosage and confidence again as the results with explainable DIL system assistance (scenario 3). We ensured that at least one week elapsed between phases, and participants were re-presented with the same cases under AI/XAI-assisted conditions without being explicitly informed that they had previously reviewed them. The workflow of these three scenarios can be found in Supplementary Fig. 2.

In these three scenarios, the accuracy of dosage recommendations was mainly evaluated by MAE (mean absolute error) between the clinician’s and the expert’s recommendations. We also used clinical agreement as an alternative metric to assess dosage differences, defined as the adjustment direction given by the doctor aligning with the expert regimen, and a dose variation within 20%. The confidence was rated on a 10-point Likert scale from 1 (not at all confident) to 10 (totally confident). See Supplementary Note 3 for questionnaire examples.

#### Faulty explanation exploration

To exclude blind belief in deep learning models and explainable algorithms, we further tested the impact of “faulty” explanation AI assistance. We generated the “faulty” explanation and conducted an exploration in 10 cases. Specifically, faulty explanations were randomly generated by shuffling feature-contribution pairs and recombining them, followed by expert review to ensure that no items overlapped with plausible but rare clinical scenarios. The clinician’s decision process was still divided into three steps, the first two of which remained to make decisions without AI assistance and with plain AI dosage assistance. In the third step, our final version DIL explanation items were replaced with synthetic faulty explanation items. Clinicians need to make decisions with AI dosage and faulty interpretation assistance as the results with faulty explanation AI assistance (scenario 4). During the process, the physician was not informed that the explanations were faulty, and all decision-making processes were the same as before. A weighted benefit based on a combination of the two indices of insulin dose accuracy and confidence was used to compare the impact of correct and faulty explanation items on doctors following Zhang K et al. and De Fauw J et al.^34,35^. There are nine situations of AI’s impact on doctors and the resulting benefit score for clinicians from the model is defined in the matrix (Supplementary Fig. 3).

### Statistics and Reproducibility

In all our studies, categorical result values were expressed as frequencies (percentages) and were compared with chi-square tests for two-sided P value. Continuous result values were expressed as mean (SD) and were compared with Mann-Whitney U test for two-sided P value. Pearson correlation was used to assess the association between feature values and their contribution effects produced by the explanation algorithms, and both the correlation coefficient (r) and two-sided P values were reported. To assess the impact of XAI assistance on performance metrics (including MAE, clinical agreement, and confidence), mixed-effects model analyses were conducted with group (junior vs. senior) and scenario as fixed effects. A p-value < 0.05 was considered statistically significant and significances were indicated as *p*  <  0.05 (*), *p*  <  0.01 (**), and *p*  <  0.001 (***).

## Results

### Overview of the dataset characteristics and proposed XAI framework

To develop and evaluate our explainable AI system, a total of 1567 inpatients with T2D encompassing 24,679 treatment days was included in our study. The demographics and clinical characteristics of patients are detailed in Table 1. The deep learning-based insulin dosage titration model demonstrated superior performance, exhibiting a Mean Absolute Error (MAE) of 0.997 in the internal dataset and 1.116 in the external dataset.

To interpret the deep-learning model, we developed an XAI framework in two phases. In the first phase, we utilized the STII to quantify the contributions of features. STII can evaluate both individual and interactive effects, making it highly suitable for complex models, such as those used in insulin dose prediction. In the second phase, we integrated expert domain knowledge into the STII calculations. Specifically, we embedded medical expertise as constraints in the LASSO regression. This step was crucial for ensuring that the model’s explanations were in line with clinical understanding.

The evaluation was carried out in three stages: statistical evaluation, expert evaluation and clinical study assessment. During the statistical evaluation, we analyzed the predictive features of our DIL framework in comparison with those of other algorithms. We also assess the consistency between our interpretation content and medical knowledge. In the expert evaluation, three endocrinologists were participated in the evaluation to compare the interpretation quality of the original STII and final STII-DIL framework. Finally, a multi-reader multi-case AI-assistance study was conducted to investigate the clinical significance of STII-DIL XAI framework.

### The predictive features analysis of STII-DIL and other algorithms

**Fig. 2:**
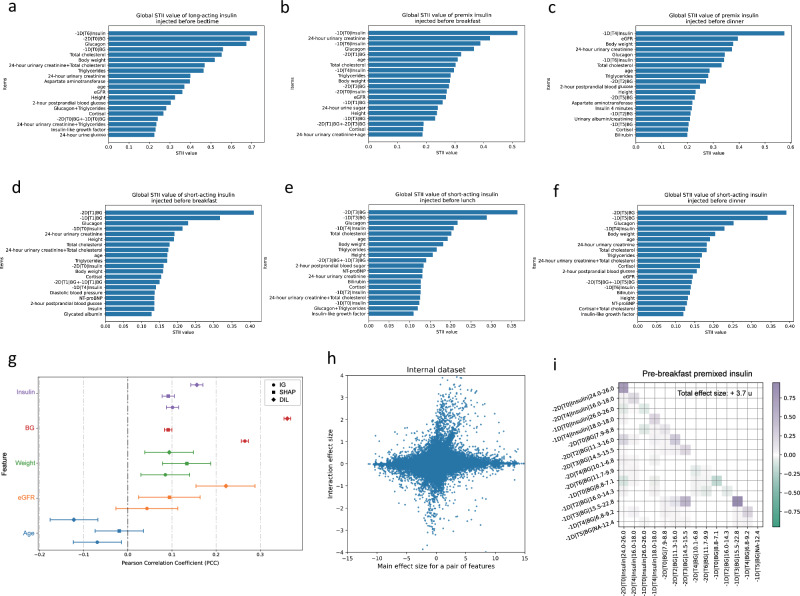
Most predictive features determined by the final version explainable algorithm. **a–f** Top 20 influential features with the highest mean absolute STII value on the internal dataset for **a** before bedtime long-acting insulin, **b** pre-breakfast premixed insulin, **c** pre-dinner premixed insulin, **d** pre-breakfast short-acting insulin, **e** pre-lunch short-acting insulin, and **f** pre-dinner short-acting insulin in the final explanation on the internal dataset. **g** Forest plot shows the PCC value for different predictive features (insulin, *n* = 19591; blood glucose, *n* = 51797; weight, *n* = 1242; eGFR, *n* = 777; Age, *n *= 1275) analyzed by three interpretability methods. Each method is represented by a unique marker (IG: circles, SHAP: squares, DIL: diamonds). Points indicate the mean PCC, and error bars represent the 95% CI of PCC values. Detailed information is provided in Supplemental Data. **h** Scatter plots of main effects of pairs of features vs interaction effect for the pair. In the internal validation dataset, treating two features as a whole in terms of their effect size on the prediction does not show a linear relationship compared to the main effect size of the two features. **i** Heat map illustrating feature effects for a patient treated with premixed insulin without oral antidiabetic medication. The color denotes predictors that decrease (green) or increase (purple) the insulin adjustment from base value. Deeper color indicates stronger contributions to the predicted insulin. -2d, two days ago; −1d, yesterday; 0 d, the current day; T0, pre-breakfast; T1, post-breakfast; T2, pre-lunch; T3, post-lunch; T4, pre-dinner; T5, post-dinner; T6, before bedtime. PCC Pearson Correlation Coefficient, eGFR estimated Glomerular Filtration Rate.

To validate the choice of our explainable algorithm, we compared its performance with other common algorithms, namely IG and SHAP. We first evaluated IG and SHAP at a population level using both internal and external datasets. Our analysis of the top 20 influential items across various insulin regimens revealed that historical insulin injection records were crucial for insulin titration in both IG and SHAP. However, these important insulin injection records did not align with the predictive insulin regimens, and BG was not significant for some regimens. This discrepancy with established medical knowledge suggests that traditional algorithms require further optimization (Supplementary Figs. 4-5). In contrast, our STII-DIL algorithm identified that historical prescribed BG records (the most relevant BG record during the insulin’s action time), same-type insulin injection records, and several patient characteristics (including age, weight, eGFR, and triglyceride levels) were most influential for predictions (Fig. 2a-f, Supplementary Fig. 6, Supplementary Data 1). The inclusion of interaction features among these top items indicates that our model effectively accounts for the interplay between different features.

To further assess the quality of different algorithms, we analyzed several features individually (Fig. 2g, Supplementary Data 1). Results showed that insulin dosage was not significantly associated with age in the SHAP algorithm and estimated glomerular filtration rate (eGFR) in the IG algorithm (P values > 0.05), which contradicted medical knowledge. In our STII-DIL algorithm, patient-related clinical features, such as age, body weight, eGFR, blood glucose (BG), and insulin injection doses were significantly correlated with multiple insulin regimen decisions, aligning with clinical practice.

Furthermore, we explored the second-order interactions provided by our algorithm. This approach allows for a nuanced exploration of interactions between features, as detailed in Fig. 2h. Specifically, features with significant interactions near the y-axis imply synergy, such as a greater interaction effect when decreasing insulin with increasing BG levels compared to their individual effects. Conversely, points near the x-axis represent minimal interactions, indicating antagonism. This observation highlights that the STII algorithm’s capability to explore the complex interactions between features. We also presented the heat map to depict the impact of both single and interactive features on insulin dosing in several cases, which align consistently with clinical knowledge at the individual level. (Fig. 2i).

In addition, to further examine feature relevance in a model-agnostic manner, we evaluated permutation importance based on the neural network model. The results suggested that previous insulin doses remained influential, showing a trend generally consistent with the STII findings (Supplementary Fig. 7).

Collectively, these findings underscore that our algorithm exhibits superior consistency with medical knowledge compared to traditional methods.

To further validate robustness, we replaced the LASSO algorithm with the random forest algorithm. The results similarly emphasized previous insulin doses and BG levels, showing a consistent but more concentrated importance pattern (Supplementary Fig. 8).

### Enhancements from DIL Integration

**Fig. 3:**
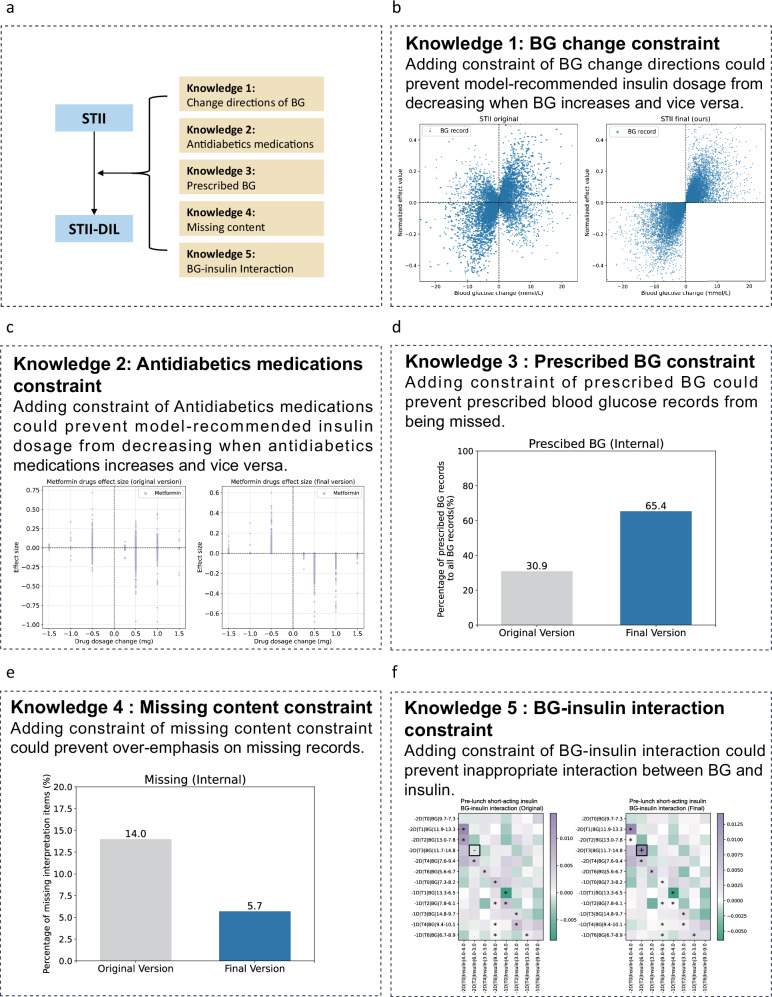
Explainable algorithm improvement with added constraints. **a** The original explainable algorithm is optimized through encoding medical knowledge and the final version was developed by adding five categories of constraints. **b** “BG change” constraint prevents insulin dose discrepancies with blood glucose changes. **c** “Antidiabetics medications” constraint prevents insulin dose discrepancies with antidiabetics medications changes. **d** “Prescribed BG” constraint encourages focus on adhering to prescribed blood glucose levels. **e** “Missing content” constraint prevents over-emphasis on missing records. **f** Explanation improvement with added “BG-insulin interaction” constraints. An example of explanation of pre-lunch short-acting insulin prediction. This patient’s pre-lunch insulin dose was reduced from 6 u to 3 u the day before, but his post-lunch blood glucose dose increased from 11.7 to 14.8 mmol/L the day before. According to medical knowledge, this BG-insulin interaction should not have a negative effect value on prediction. The original version of the algorithm assumes that the effect value is negative, which is wrong (Left). After adding the constraint, the final version of the algorithm considers this effect is positive (Right).

To align the STII algorithm with practical clinical insights, we implemented a DIL framework, integrating expert-guided domain knowledge into the algorithm’s development (Figs. 1b,3a).

We first conducted a statistical evaluation to assess the interpretive quality improvement post-DIL integration. Notably, after applying the BG change constraint, we observed a significant reduction in the number of data points in the second quadrant, where a decrease in BG positively correlates with insulin dosage, and in the fourth quadrant, where an increase in BG negatively affects the dosage (Fig. 3b and Supplementary Fig. 9). This suggests a marked reduction in discrepancies between predicted insulin dosages and observed BG changes. Similarly, the integration of constraint related to antidiabetic medications refined the model’s accuracy (Fig. 3c and Supplementary Fig. 10), Adding the prescribed BG constraint increased the proportion of prescribed records, rising, from 30.9% to 65.4% in the internal dataset and from 26.0% to 43.5% in the external dataset (Fig. 3d and Supplementary Fig. 11). The effect of missing content constraint is shown in Fig. 3e and Supplementary Fig. 12. Moreover, incorporating the BG-insulin interaction constraint helped correct previously unreasonable influences in insulin dosage predictions (Fig. 3f and Supplementary Fig. 13).

**Fig. 4:**
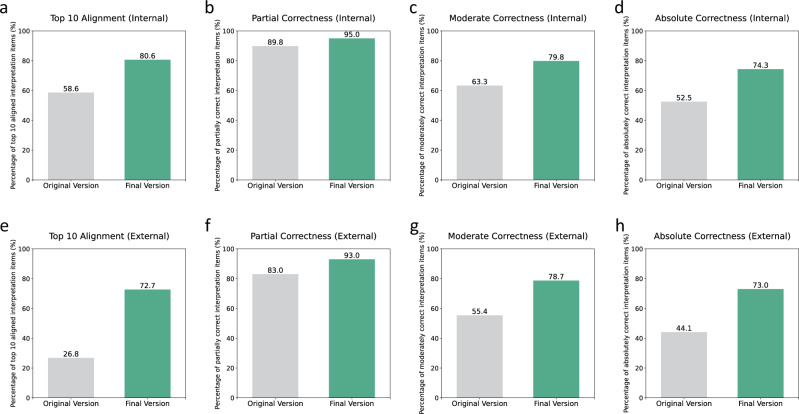
Performance evaluation between the original and final version by experts. Comparison of original and final explanation versions on (**a–d**) an internal dataset (*n* = 20 regimens, 40 insulin points) and (**e–h**) an external dataset (*n* = 20 regimens, 46 insulin points) (**a**,**e**) Top ten alignment rate. (**b**,**f**) Partial correctness (with correct features) rate. (**c**,**g**) Moderate correctness (with correct features and directions) rate. (**d**,**h**) Absolute correctness (with correct features, directions, and effect size).

To further validate the effectiveness of the DIL methodology, we performed an expert evaluation on 40 cases. The evaluation demonstrated significant improvements in the model’s alignment with clinical practice: the top ten alignment rate improved from 58.6 to 80.6%, the partial correctness rate from 89.8 to 95.0%, the moderate correctness rate from 63.3 to 79.8%, and the absolute correctness rate from 52.5 to 74.3% (Fig. 4, Supplementary Fig. 14, and Supplementary Table 1, see methods for detailed metric definitions). These enhancements were consistent across both internal and external datasets.

These findings confirm that the DIL approach significantly enhances the medical alignment and correctness of our explainable AI model, demonstrating its efficacy in improving algorithmic predictions in line with clinical knowledge.

### Multi-reader multi-case AI-assistance assessment

**Fig. 5:**
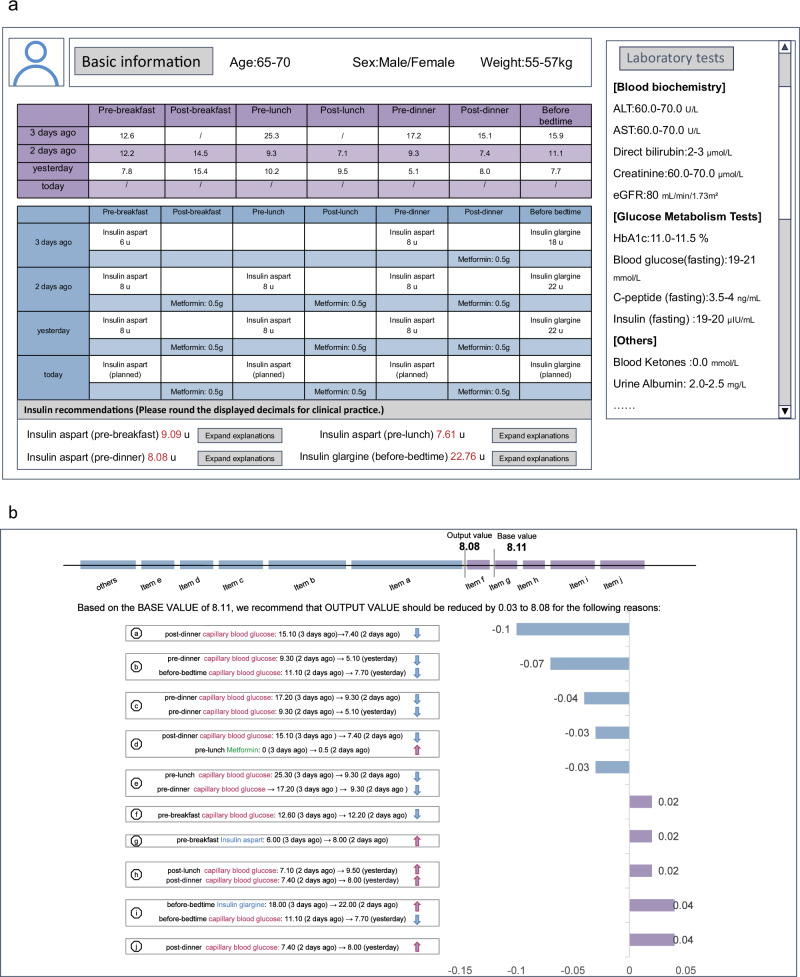
Interface Showcase Diagram. **a** Patient record and insulin recommendations. **b** Explanations of insulin dosage. Patient data has been de-identified and presented in ranges to ensure privacy and confidentiality.

The insulin predictive and explainable model was deployed at Zhongshan Hospital in December 2022 to provide real-time insulin dosage recommendations and explanations through its interface (Fig. 5).

Furthermore, we conducted an AI-assistance study to explore the potential of our explainable system as a clinical decision support tool. Records from forty patients were included in this study (Supplementary Table 1). Eight clinicians with different levels of clinical experience were recruited and assigned to two groups: junior clinicians with 1-3 years of clinical experience (*n * =  4), and senior clinicians with 4–7 years of clinical experience (*n* = 4). We compared the clinicians’ decision accuracy and confidence in three scenarios: without AI assistance, with plain AI dosage assistance, and with AI dosage and XAI assistance (Fig. 1d and Supplementary Fig. 2).

**Fig. 6:**
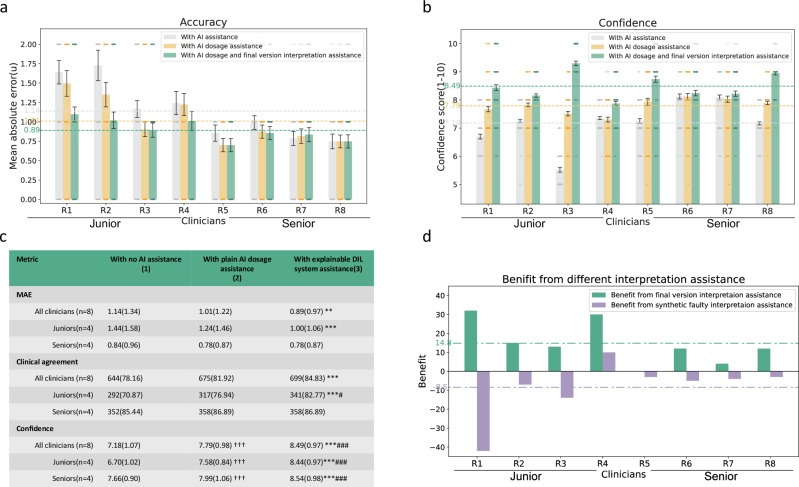
Performance of clinicians in different AI-assistance scenarios. **a–c** Comparison of dosage accuracy and decision confidence of clinicians with no AI assistance (scenario 1), with plain AI dosage assistance (scenario 2), and with explainable DIL system assistance (scenario 3). **a** recommended insulin error (MAE) and **b** confidence score (1-10) in the decision of 8 clinicians in different scenarios. Bar graphs indicate the mean ± s.e.m. R, reviewer. **c** MAE, clinical agreement (same direction, dose difference ≤20%), and confidence score (1-10) of each subgroup in different scenarios. The two-sided Mann-Whitney U test was used to compare the MAE of dosage and confidence score in different scenarios. The two-sided χ2 test was used to compare the clinical agreement in different scenarios. †Significant difference between the scenario 2 and 1, †p < 0.05, ††p < 0.01, †††p < 0.001; *Significant difference between the scenario 3 and 1, *p < 0.05, **p < 0.01, ***p < 0.001; #Significant difference between the scenario 3 and 2, #p < 0.05, ##p < 0.01, ### p < 0.001. Data are n (%) or mean (s.d.). MAE, mean absolute error. **d** Clinicians’ benefit from final version DIL explanation assistance and synthetic faulty explanation assistance. Benefit was calculated using dosage accuracy and decision confidence (see Supplementary Fig. 14 for a detailed definition.).

We first assessed our XAI’s influence on the clinicians’ decision accuracy using both MAE and “clinical agreement” (defined as the same direction with dose difference ≤20%) by taking the dosage recommended by the expert panel as reference. We found that there was slight but no significant improvement in clinicians’ decision accuracy between clinicians with no AI assistance and with plain AI dosage assistance (Fig. 6c). While supported by our XAI system, clinicians achieved a lower mean absolute error (MAE) of 0.89 U compared to 1.14 U with no AI assistance (*P* < 0.01) (Fig. 6a). Additionally, the percentage of “clinical agreement” of clinicians was 84.83% with XAI assistance, higher than no AI assistance (*P* < 0.001). Besides, compared with plain AI dosage assistance (MAE: 1.01; clinical agreement: 81.92%), our XAI system slightly improved clinician’s decision accuracy, but the difference was not statistically significant. Subgroup analysis revealed a significant enhancement in the decision accuracy among junior clinicians rather than senior clinicians, suggesting that clinicians with less experience benefited more from our XAI system.

Next, we assessed the influence of our XAI on the clinicians’ decision confidence, rated on a 10-point Likert scale (1= not at all confident; 10= totally confident). The mean confidence score per clinician in each scenario is illustrated in Fig. 6b. Compared with the confidence score of 7.18 when no AI assistance, the clinicians’ confidence with both plain AI dosage (score: 7.79) and XAI system assistance (score: 8.49) was significantly increased (both *P* < 0.001). Subgroup analyses revealed improved confidence in both junior and senior clinicians (Fig. 6c).

Detailed comparisons of the decision accuracy and confidence between junior and senior clinicians across the three scenarios are provided in Supplementary Fig. 15, which show the differential benefits of the XAI system for each group.

Furthermore, considering the mere presence or absence of explanations, rather than their quality, also has potential impact on clinicians’ decision^27^, we designed “faulty” explanations which were synthetic and inconsistent with medical knowledge, and we further explore their effect on clinicians’ decisions. We found that previously observed benefits (see Supplementary Fig. 3 for definition) of DIL assistance were weakened and even reversed when the XAI explanation was faulty. Figure 6d revealed that junior doctors were particularly susceptible to underperformance if a fault explanation occurred in this context. This result demonstrated that low-quality explanation may have detrimental consequences and disrupt clinical workflow, underscoring the importance of reasonable explanations.

## Discussion

In this study, we developed a second-order explainable algorithm framework STII-DIL incorporated with expert knowledge to interpret deep-learning-based insulin titration model. Compared to common first-order algorithms, the framework provides additional explanations for interaction factors based on the second-order STII algorithm and reduces unjustified post hoc explanations by incorporating medical knowledge. The final version of the DIL algorithm showed knowledge-aligned results on both internal and external validation sets and performed better than the original version. Furthermore, we conducted an AI-assistance study on such an XAI system in insulin titration and indicated that our system boosted the decision accuracy and confidence of clinicians.

Recognizing the intricate interplay between features in the complex physiological environment of diabetes, we first opted for the STII, a second-order interpretation algorithm, to elucidate our insulin dosage predictive model. Our STII-DIL algorithm offers distinct advantages compared to other interpretability methods, such as gradient-based or attention-based approaches. Gradient-based and other similar first-order approaches typically capture only the relationships between input and output, often overlooking the complex feature interactions and non-linearities inherent in deep learning models. This can lead to insufficient explanations, particularly in cases where higher-order feature interactions are critical, such as in diabetes treatment. Attention mechanisms, while capable of capturing some of these interactions, tend to produce unstable explanations, as attention weights can be sensitive to fluctuations during the training process^36^ and their use is limited by the network structure, making their interpretability less reliable in complex networks. In contrast, STII-DIL is built upon the Shapley Taylor interaction index, which is theoretically grounded and capable of accurately modeling interactions between features, ensuring consistent and fair explanations. Compared to Shapley interaction, which is used in some research, STII has the advantage that it satisfies the efficiency axiom, a necessary condition met by classical algorithms in the Shapley value series^28^. In conclusion, our choice of the STII model provides a more comprehensive understanding of the insulin decision-making process and is adept at explaining complex physiological environments than the majority of current studies that focus on single-feature first-order interpretable algorithms.

While second-order post-hoc explanation holds promise, our findings on the original version of the STII algorithm suggested that direct application of the algorithm occasionally produced inappropriate explanations. Recent researches highlighted incorporating human input within medical AI systems will be beneficial and could guarantee the safety-critical nature of medical domain^22,37,38^. The application of knowledge graphs has shown potential in increasing interpretability^39,40^. However, for problems that do not have a complete body of knowledge, it remains challenging to bring in physician knowledge while ensuring model interpretability. Addressing this, we adopted a ‘DIL’ methodology by integrating human domain knowledge into the algorithm. Unlike previous human-in-the-loop studies, which were primarily focused on feature selection and concept summarization^41–45^, our approach actively involves experts’ domain knowledge in the iterative development process of the algorithm. Results showed a significant reduction in explanation items inconsistent with medical knowledge by encoding expert knowledge into the constraints of the optimization process, indicating our framework can effectively narrow the solution space to areas deemed acceptable by medical professionals.

In contrast to previous studies that mainly focused on model performance with only preliminary measures of explainability^46–50^, our study conducted a series of assessment to guarantee the performance of our explanations. Firstly, we evaluated the explainable algorithm in the internal and external validation set and the results suggested that the algorithm is reasonable and robust at both population and individual levels. Secondly, as Gulum MA et al. suggested^51^, metrics used to measure explanation quality should be designed with clinicians involved. Therefore, we used a combination of statistical and expert evaluation to confirm the improvement of the final explanation version, both in terms of correctness and alignment. Thirdly, considering that real-life medical practice is more likely to involve AI-human collaboration than AI-versus-human comparison^52^, four scenarios (with no AI assistance, with plain AI dosage assistance, with explainable DIL system assistance, and with synthetic faulty explanation assistance) in real-world insulin decision were designed to explore the role of explainability in AI-human collaboration. Notably, we found that providing plain AI dose recommendations contributed little to improving the accuracy of clinicians, whereas AI-assisted models with reasonable explanations could significantly improve the accuracy. This suggests that doctors may be unwilling to adopt assistance from a “black-box” tool. This may also suggest that, in some scenarios, explanations are indispensable for the model to work rather than an add-on. As a result, XAI can increase the adoption of AI assistants since it enhances clinicians’ confidence in their decisions. Similar to previous studies^53^, our study found that less experienced clinicians had benefited more from the explainable framework, suggesting that our framework is expected to improve healthcare quality for a wide range of junior clinicians. In contrast, the improvement from plain AI to XAI assistance among senior clinicians was modest in accuracy but significant in confidence, likely because the model was trained on expert-derived insulin labels and the DIL constraints were aligned with senior-level reasoning^54,55^. Besides, our results suggested XAI had potential to increase the adoption of AI assistants since it enhances physicians’ confidence in their decisions, which is consistence with the other research^56,57^. Finally, the exploration results of synthetic faulty explanations demonstrated that low-quality explanation may have detrimental consequences and disrupt clinical workflow, underscoring the importance of incorporating physician knowledge to ensure the accuracy of explanation. To mitigate the potential risks of explanation uncertainty, only the top 10 explanations identified by the algorithm are displayed, with optional hiding functions and continuous institutional monitoring to ensure safe and accountable deployment.

Our study offers several innovations. Firstly, we developed a framework that harmonized the explainable algorithm’s mathematical optimization with endocrinology expertise, so as to provide accurate and medically relevant explanation in clinical decision-making. Secondly, we designed a feasible human-machine collaboration pattern that can actively and effectively involve doctors in the model construction. The loop process design helps to align algorithm knowledge to expert level gradually. Thirdly, we adopted an interpretative assessment process that incorporates multiple perspectives, such as statistical, expert, and clinical assessments to ensure comprehensiveness, and multiple datasets, such as internal and external datasets to ensure robustness. Consequently, our study has the potential to generate a transparent and personalized AI-assisted support system that uncovers “black-box” challenges in insulin titration for T2D patients.

Our study has several limitations. First, our data were collected from individuals of various ethnicities in China and the generalization of the AI to other ethnicities needs to be further investigated. Second, the doctors participating in the study were all from China hence their clinical practices might be similar, which limits the extrapolation of our conclusions. Third, considering medicine is constantly evolving, the constraints we have incorporated so far can only represent the current stage of knowledge. The DIL framework may be iteratively refined by incorporating updated clinical guidelines, expert consensus, clinician feedback, and multicenter data, ensuring sustained interpretability and clinical relevance as medical knowledge evolves. Finally, the AI-human collaboration study followed a fixed scenario order rather than randomization, which might have introduced minor order effects despite the one-week interval between phases.

## Conclusion

In summary, this study has provided a feasible framework named DIL for explaining the deep learning model by leveraging the strengths of both specialist knowledge and AI explainable algorithms. The insulin dosage titration model with DIL-improved explanation can assist junior doctors in diabetes management. We expect this approach will ultimately facilitate the development of efficient and transparent clinical decision support tools, improve the accessibility of healthcare resources, and might inform work in other areas of complex disease management.

### Reporting summary

Further information on research design is available in the Nature Research Reporting Summary.

## Supplementary information

Supplementary material

Description of Additional Supplementary files

Supplementary data1

Supplementary data2

Supplementary data3

## Data Availability

The source data for Figs. 2a–f, g, i, 3b–f, 4, and 6 are provided in Supplementary Data 1, while the source data for Fig. 2h is available in Supplementary Data 2. Because the raw dataset is patient electronic health records, it is not publicly available. De-identified data may be obtained from the corresponding authors upon reasonable request and with the required institutional approvals. Because the raw dataset consists of patient electronic health records, it is not publicly available. De-identified data can be accessed from the corresponding authors upon reasonable request and subject to the required institutional approvals and data use agreements. Requests for data access will be reviewed within approximately 4-6 weeks. Access is granted solely for research purposes and the data may not be redistributed or used for commercial applications.

## Code availability

The source code for the developed DIL algorithm and a complete description of the required runtime environment are provided via a dedicated public repository on GitHub (URL: https://github.com/962086838/Explainable-DeepLearning-framework-incorporating-medical-knowledge-for-insulin-titration-in-diabetes). The code can also be found on: 10.5281/zenodo.18217103^58^. This ensures reproducibility of our findings.
