# Supplementary material for: Explainable deep learning framework incorporating medical knowledge for insulin titration in diabetes
Source: Commun Med (Lond). 2026 Feb 26;6:192. doi: 10.1038/s43856-026-01449-1 (PMC13062103; doi:10.1038/s43856-026-01449-1)
Supplement: Supplementary file 1 — Supplementary material [file 43856_2026_1449_MOESM1_ESM.pdf]

Supplementary information

Contents

Supplementary Figure 1. Explanation development. 2
Supplementary Figure 2. Study design of AI-assistance study. 3
Supplementary Figure 3. Weight matrix of the benefit score. 4
Supplementary Figure 4. Top 20 feature contributions of IG algorithm. 5
Supplementary Figure 5. Top 20 feature contributions of SHAP algorithm. 6
Supplementary Figure 6. Top 20 feature contributions of STII-DIL algorithm on the external dataset. 7
Supplementary Figure 7. Top 20 feature of the permutation importance results. 8
Supplementary Figure 8. Top 20 feature of the RF-based STII model. 9
Supplementary Figure 9. Explanation improvement with added "BG change" constraints on the external dataset. 10
Supplementary Figure 10. Explanation improvement with added "antidiabetic medications" constraints on the internal (a-f) and external (g-n) datasets. 11
Supplementary Figure 11. Explanation improvement with added "Prescribed BG" constraints on the external dataset. 12
Supplementary Figure 12. Explanation improvement with added "Missing content" constraints on the external dataset. 13
Supplementary Figure 13. Explanation improvement with added "BG-insulin interaction" constraints on the external dataset. 14
Supplementary Figure 14. Expert evaluation results of alignment between the original and final version. 15
Supplementary Figure 15. Comparisons of decision accuracy and confidence between junior and senior clinicians. 16
Supplementary Table 1. Characteristics of datasets in human evaluations. 17
Supplementary Note 1. Questionnaire 1: Doctor in the loop 18
Supplementary Note 2. Questionnaire 2: Expert evaluation 19
Supplementary Note 3. Questionnaire 3: AI-assistance study 20

Supplementary Figure 1. Explanation development.

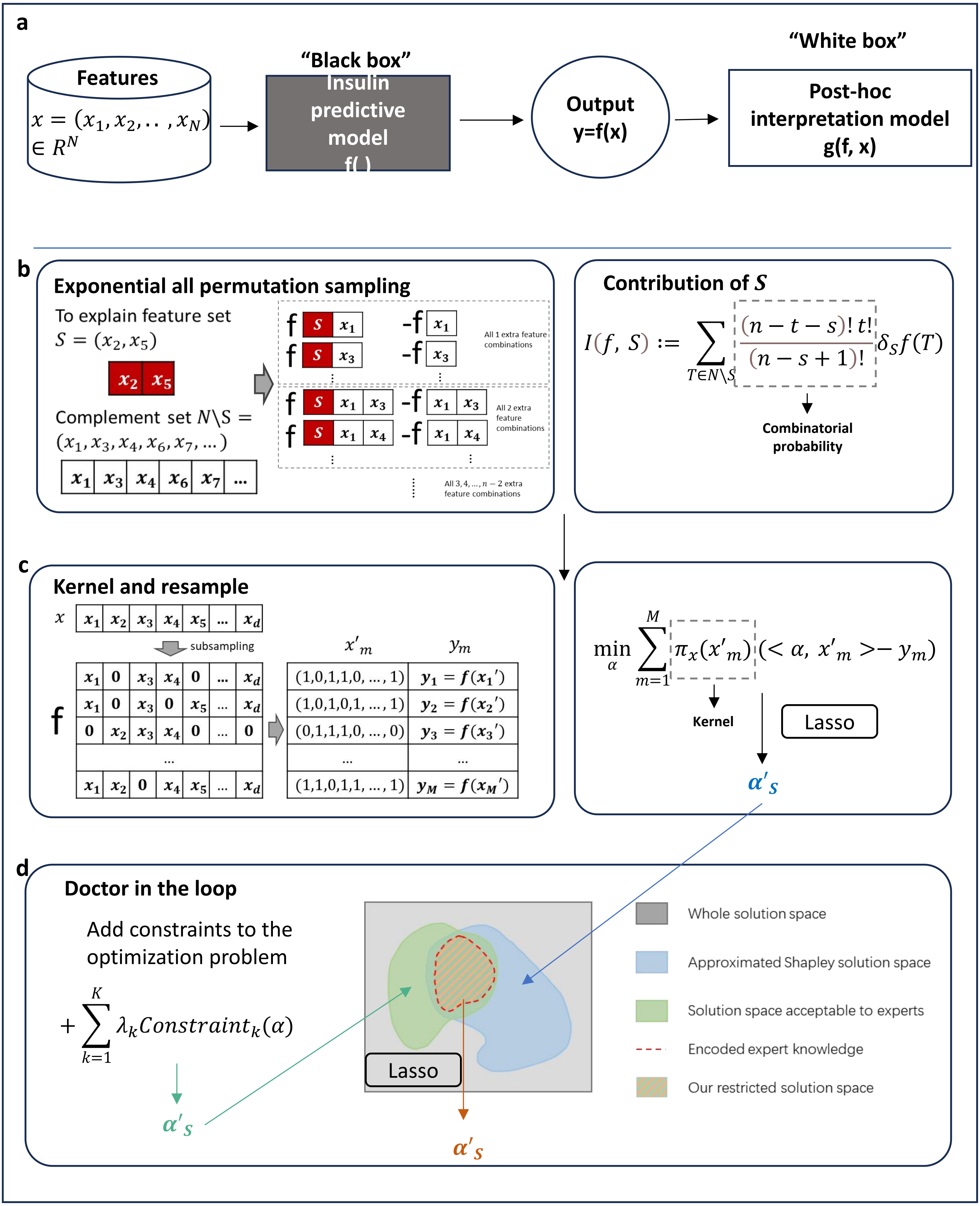

(a) Overview of the modeling process. **(b)** Interpret the effect of interaction factors. **(c)** Kernel and resample acceleration algorithm. **(d)** Embed expert knowledge into explanations.

Supplementary Figure 2. Study design of AI-assistance study.

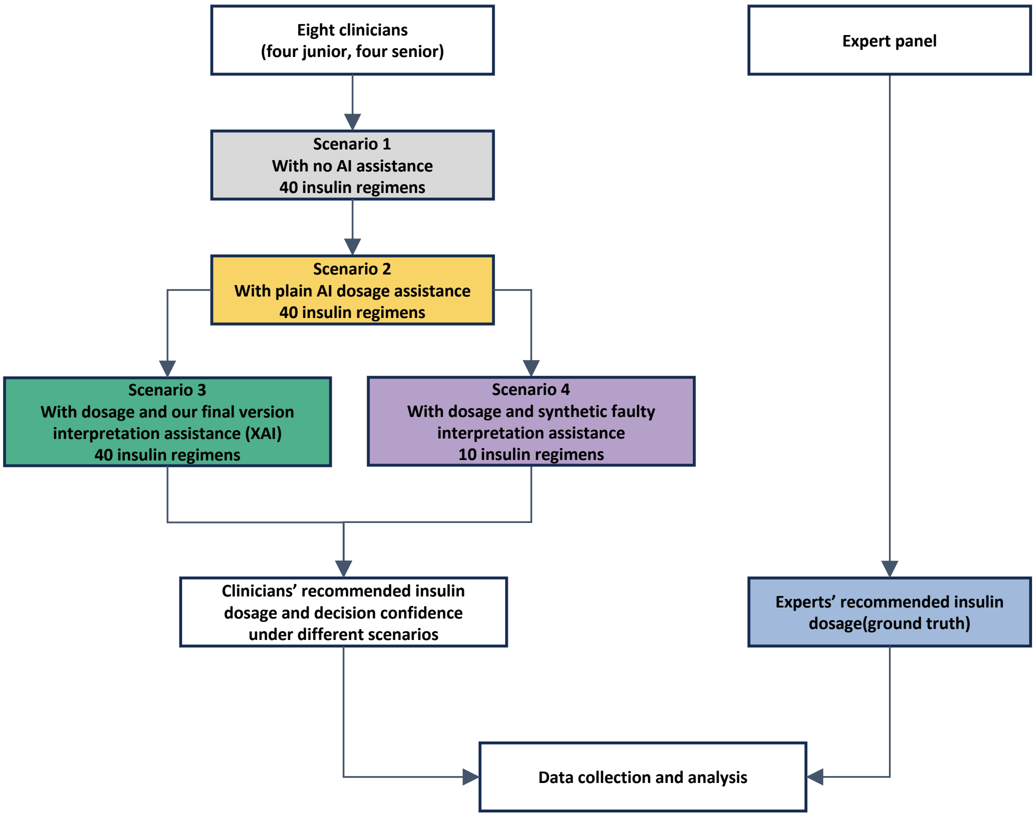

Eight clinicians consist of four junior clinicians and four senior clinicians participated in this study. They were asked to give their recommended insulin dosage and confidence scores (1-10) of 40 insulin regimens with no AI assistance (scenario 1), with plain AI dosage assistance (scenario 2), and with explainable DIL system assistance (scenario 3), respectively. They also need to give their recommended insulin dosage and confidence scores (1-10) of 10 insulin regimens with synthetic faulty explanation assistance (scenario 4). Expert panel also provided recommended insulin dosages of these 40 insulin regimens without any AI assistance, and the experts' dosages were regarded as ground truth.

Supplementary Figure 3. Weight matrix of the benefit score.

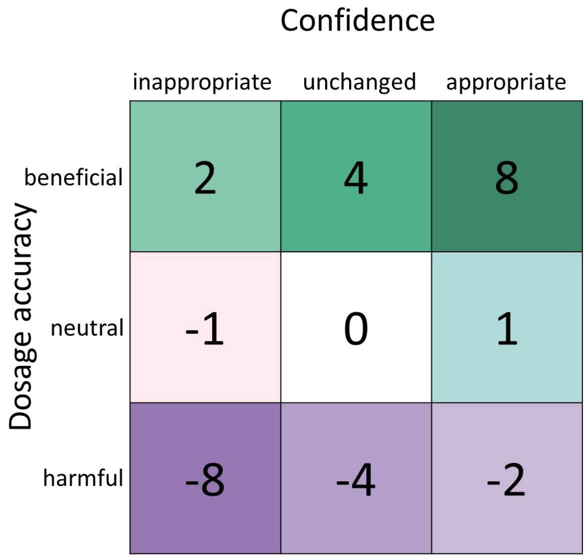

We designed a matrix that combined dosage-benefit and confidence-benefit together to assess the benefits of the explainable system to doctors, using the AI dosage-assisted scenario as a reference. The dosage benefit was categorized into three levels: beneficial, neutral, or harmful, based on the change in doctor dose accuracy. Similarly, the confidence benefit was categorized into three levels: appropriate, unchanged, or inappropriate, based on the change in doctors' confidence in decision-making. We assigned different weights to the nine scenarios based on their clinical impact. Specially, doctors gained 8 points if their dose recommendation accuracy increased with the aid of the explainable system and their confidence increased, while they lost 8 points if their dose accuracy decreased and their confidence in the inappropriate dose increased. The final benefit to the doctor was calculated as the weighted average of the benefits for all scenarios.

Supplementary Figure 4. Top 20 feature contributions of IG algorithm.

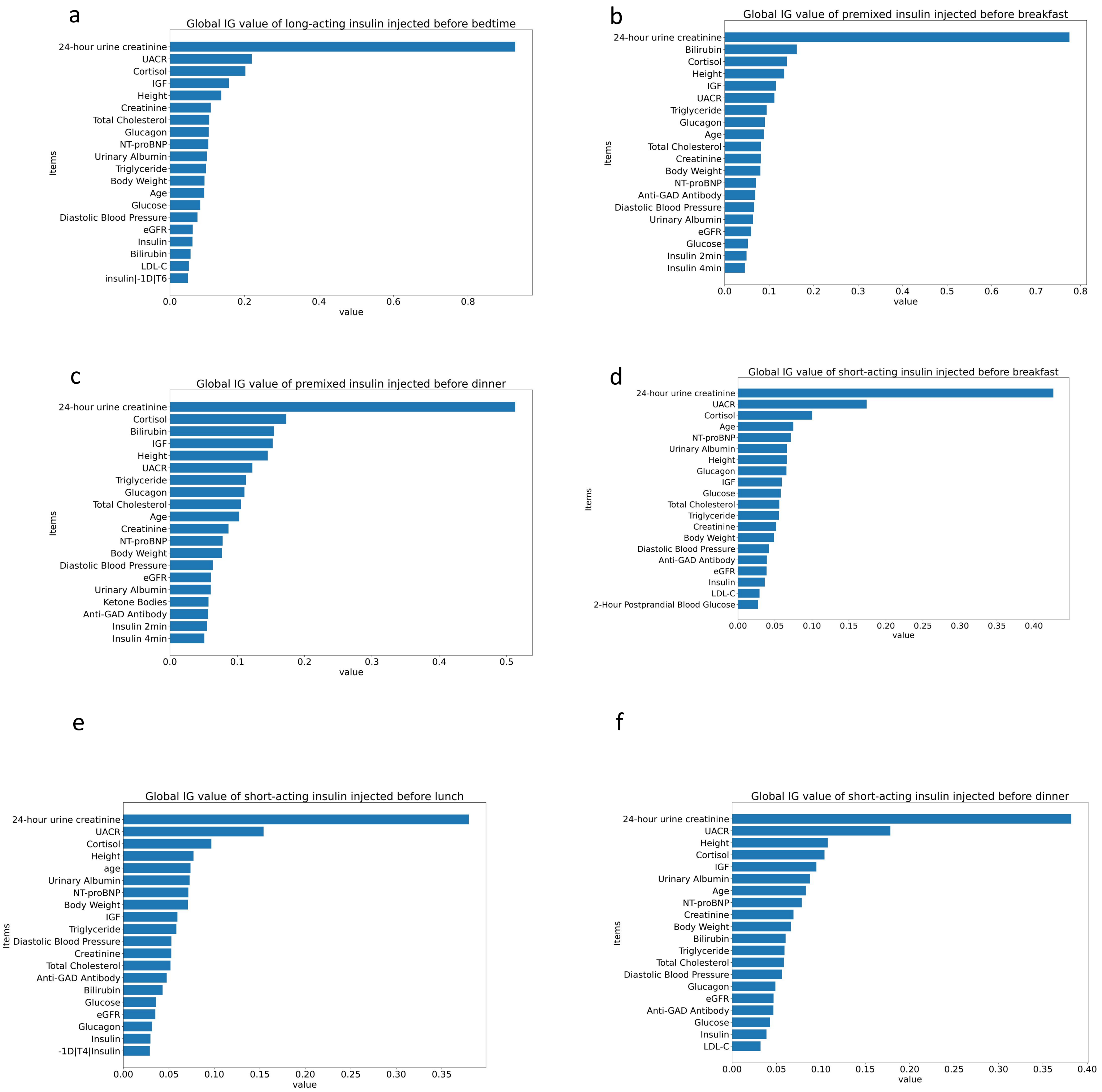

Top 20 features with the highest mean absolute contribution value for (**a**) before bedtime long-acting insulin, (**b**) pre-breakfast premixed insulin, (**c**) pre-dinner premixed insulin, (**d**) pre-breakfast short-acting insulin, (**e**) pre-lunch short-acting insulin, and (**f**) pre-dinner short-acting insulin in the final explanation on the internal dataset.

Supplementary Figure 5. Top 20 feature contributions of SHAP algorithm.

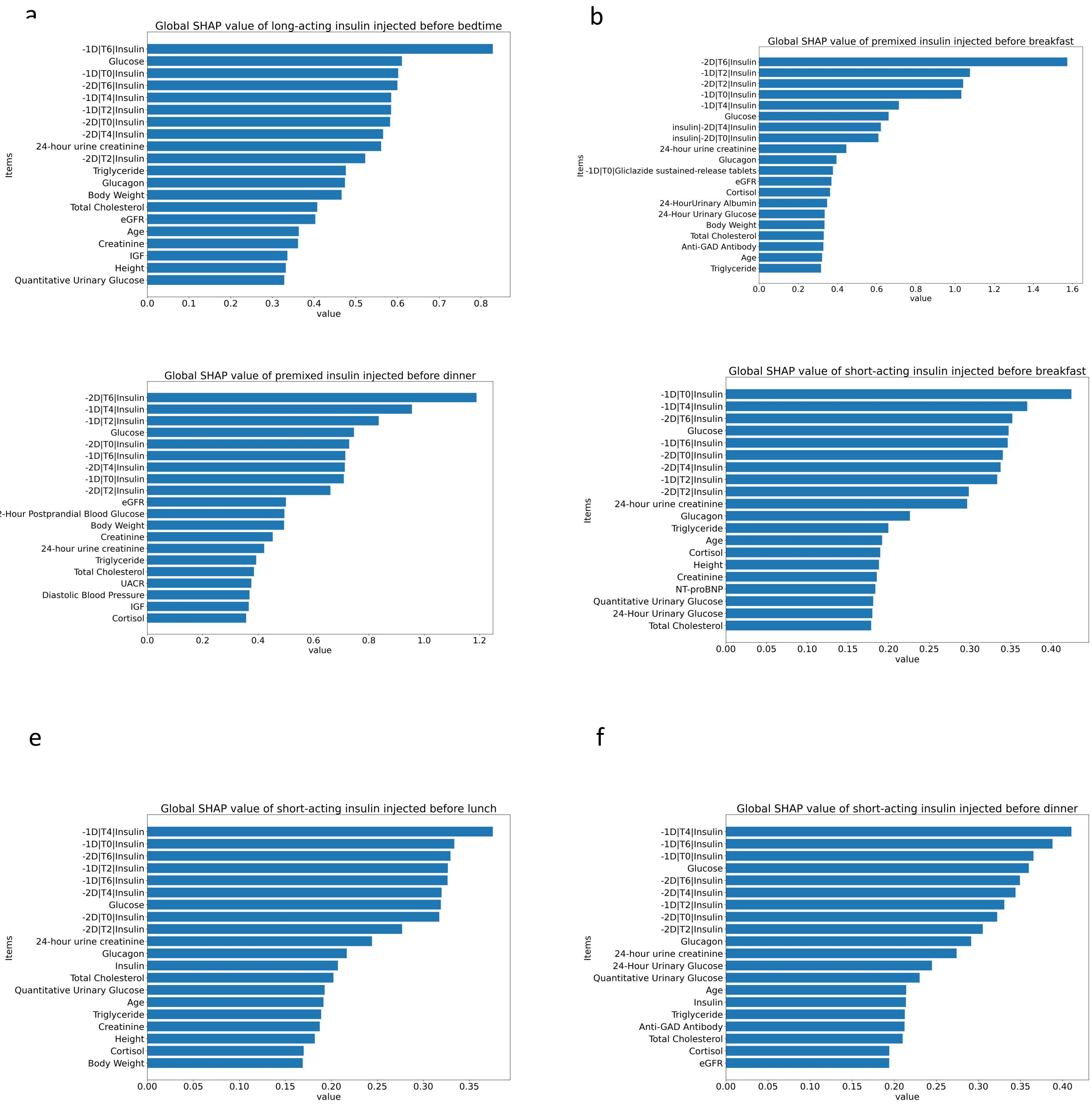

Top 20 features with the highest mean absolute contribution value for (**a**) before bedtime long-acting insulin, (**b**) pre-breakfast premixed insulin, (**c**) pre-dinner premixed insulin, (**d**) pre-breakfast short-acting insulin, (**e**) pre-lunch short-acting insulin, and (**f**) pre-dinner short-acting insulin in the final explanation on the internal dataset.

Supplementary Figure 6. Top 20 feature contributions of STII-DIL algorithm on the external dataset.

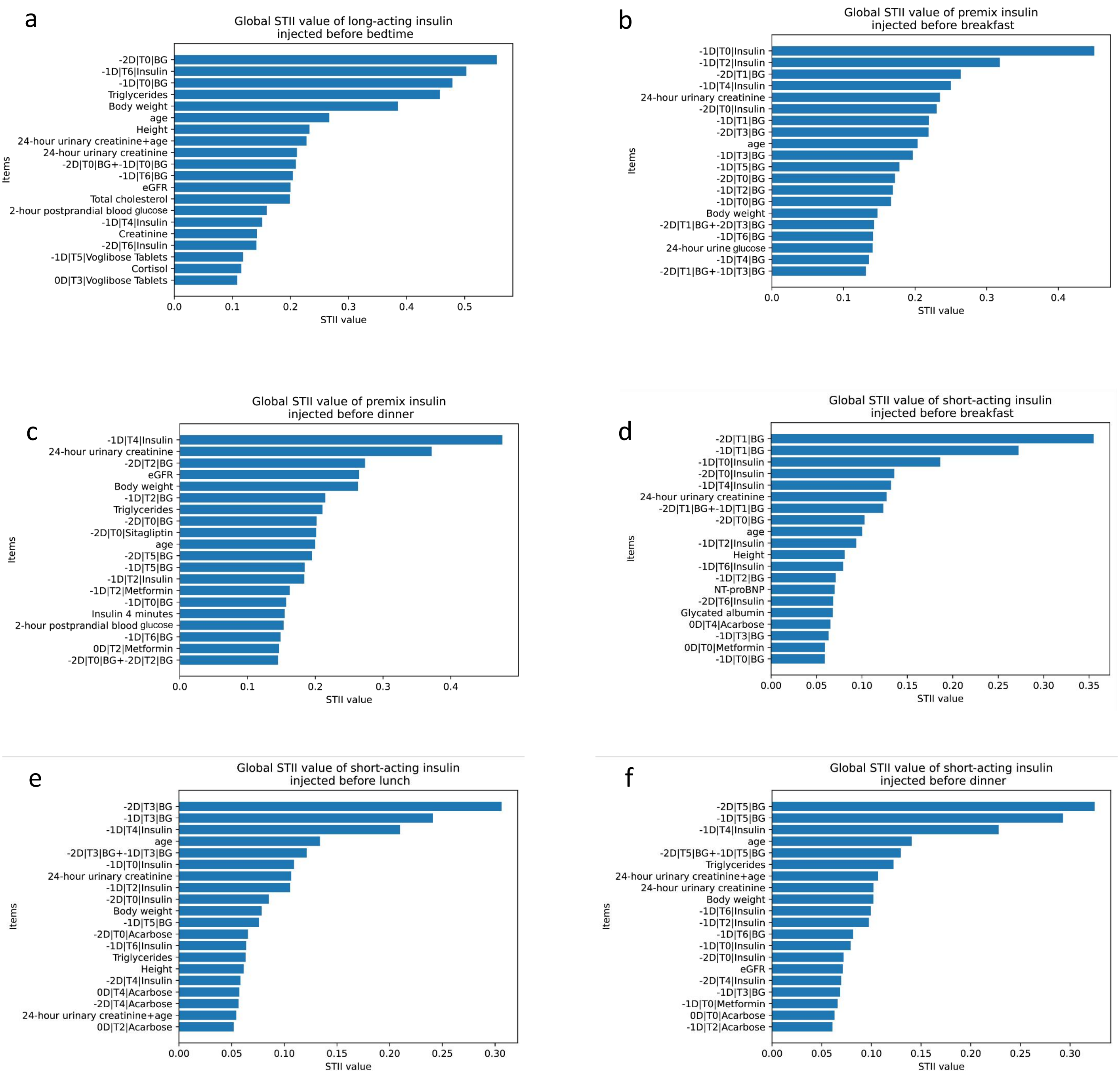

Top 20 features with the highest mean absolute STII value for (**a**) before bedtime long-acting insulin, (**b**) pre-breakfast premixed insulin, (**c**) pre-dinner premixed insulin, (**d**) pre-breakfast short-acting insulin, (**e**) pre-lunch short-acting insulin, and (**f**) pre-dinner short-acting insulin in the final explanation on the internal dataset.

Supplementary Figure 7. Top 20 feature of the permutation importance results.

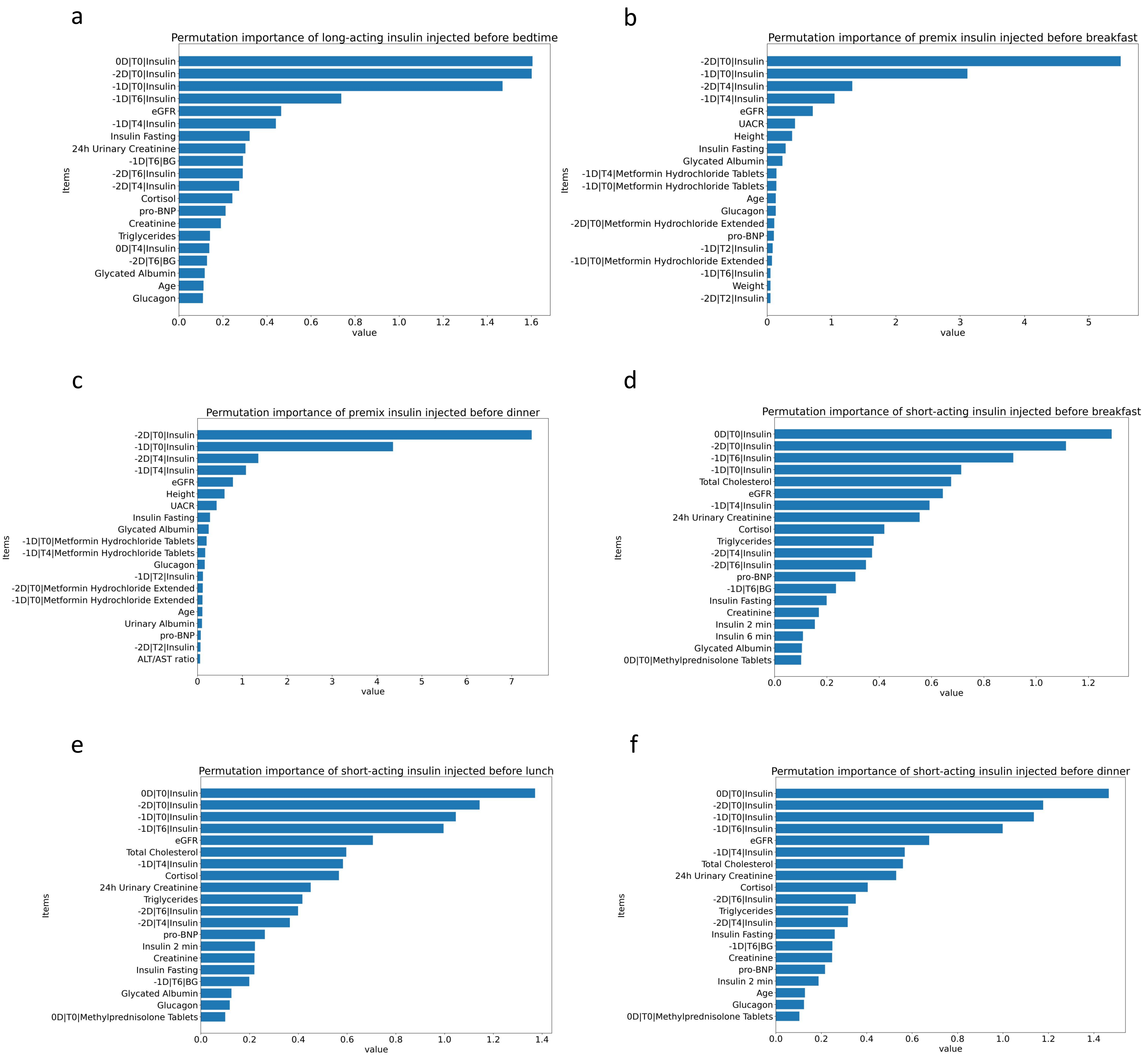

Top 20 features with the highest mean absolute contribution value for (**a**) before bedtime long-acting insulin, (**b**) pre-breakfast premixed insulin, (**c**) pre-dinner premixed insulin, (**d**) pre-breakfast short-acting insulin, (**e**) pre-lunch short-acting insulin, and (**f**) pre-dinner short-acting insulin in the final explanation on the internal dataset.

Supplementary Figure 8. Top 20 feature of the RF-based STII model.

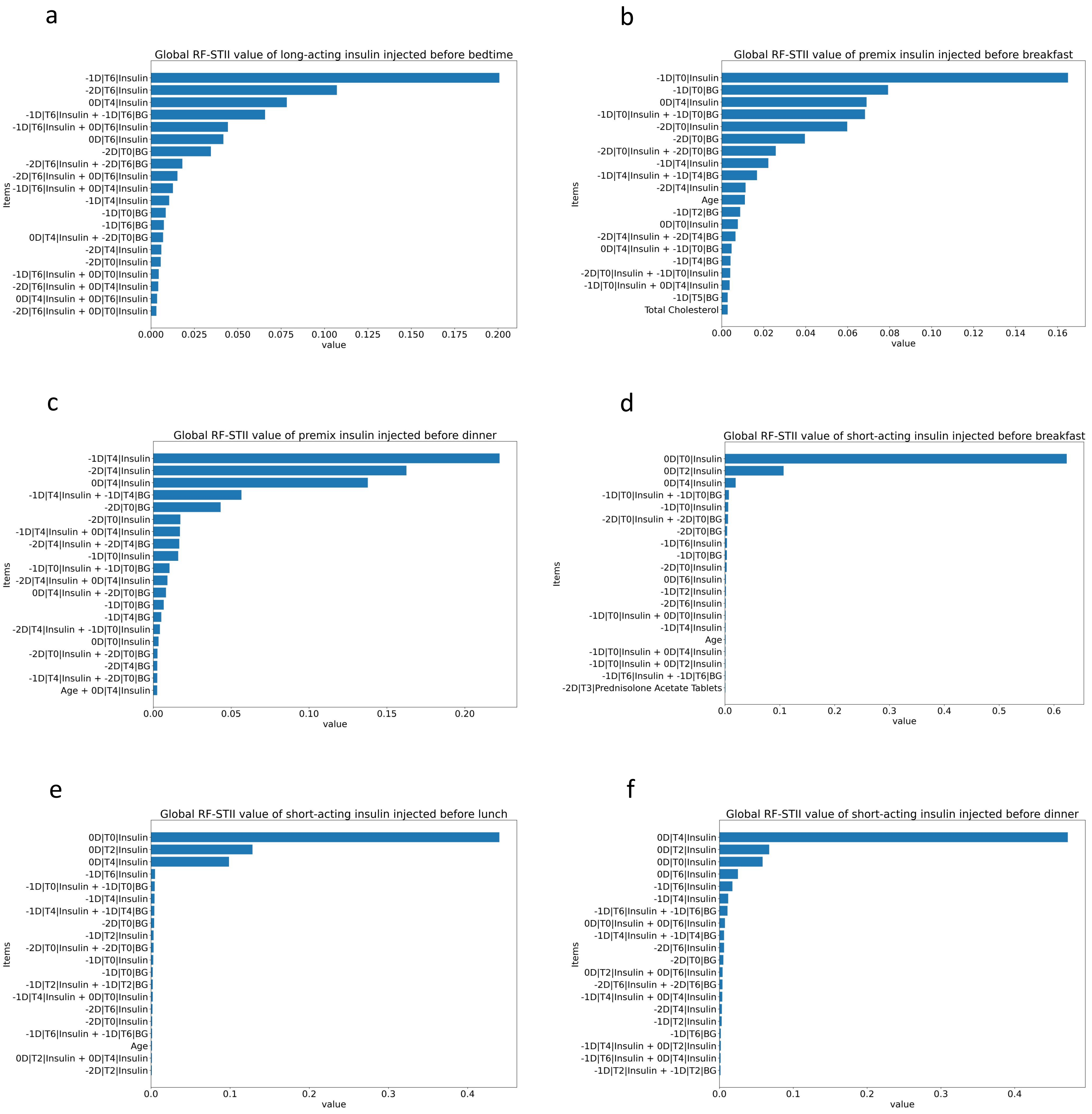

Top 20 features with the highest mean absolute contribution value for **(a)** before bedtime long-acting insulin, **(b)** pre-breakfast premixed insulin, **(c)** pre-dinner premixed insulin, **(d)** pre-breakfast short-acting insulin, **(e)** pre-lunch short-acting insulin, and **(f)** pre-dinner short-acting insulin in the final explanation on the internal dataset.

Supplementary Figure 9. Explanation improvement with added “BG change” constraints on the external dataset.

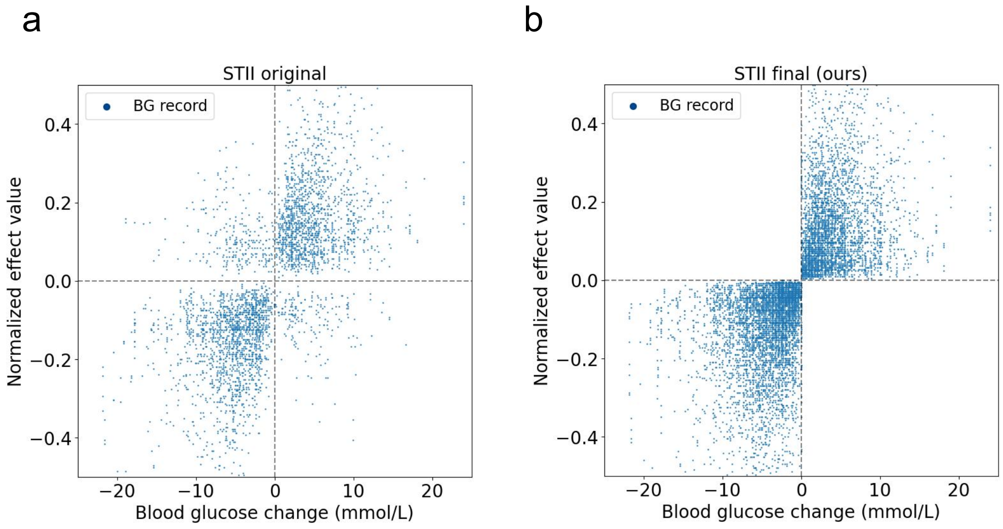

(a) the original version of explanation. (**b**) the final version of the explanation.

Supplementary Figure 10. Explanation improvement with added "antidiabetic medications" constraints on the internal (a-f) and external (g-n) datasets.

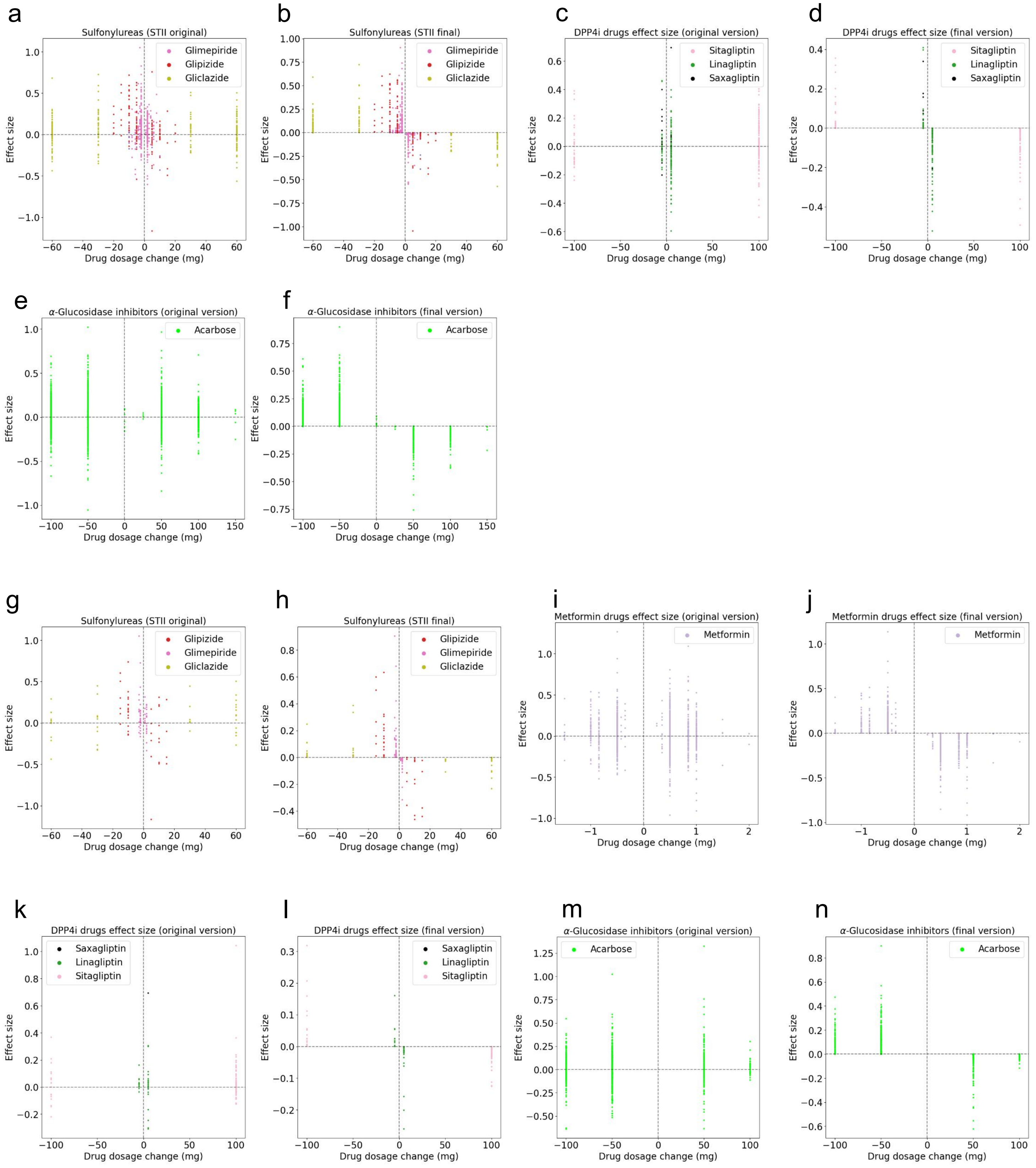

Supplementary Figure 11. Explanation improvement with added "Prescribed BG" constraints on the external dataset.

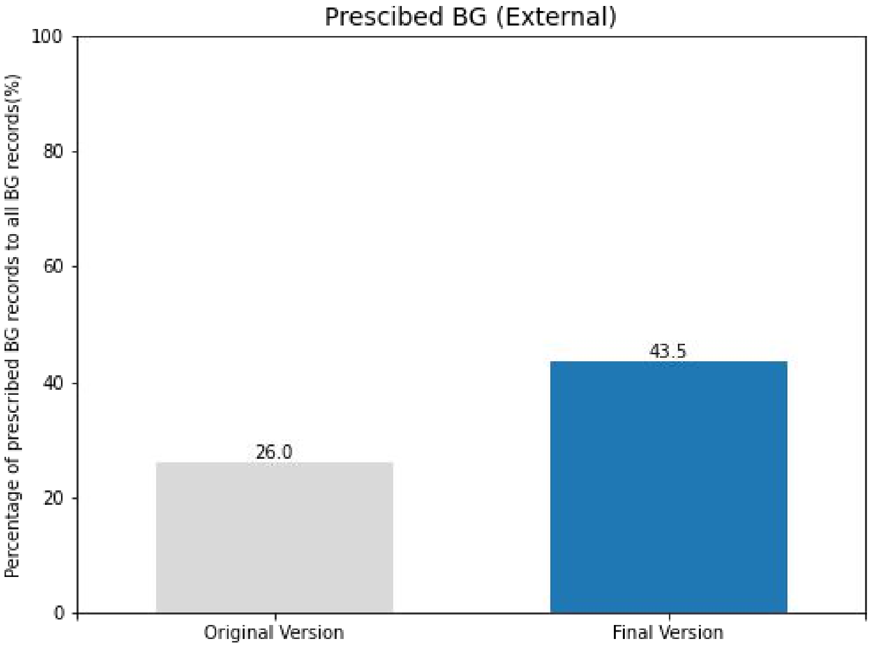

Adding "Prescribed BG" constraint encourages focus on adhering to prescribed blood glucose levels on the external dataset.

Supplementary Figure 12. Explanation improvement with added "Missing content" constraints on the external dataset.

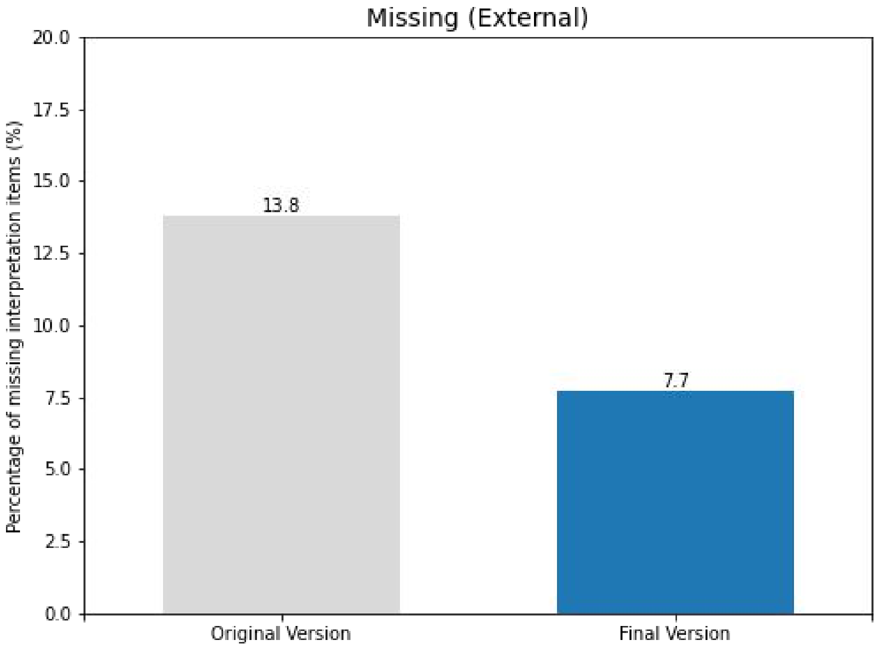

Adding "Missing content" constraint prevents over-emphasis on missing records.

Supplementary Figure 13. Explanation improvement with added "BG-insulin interaction" constraints on the external dataset.

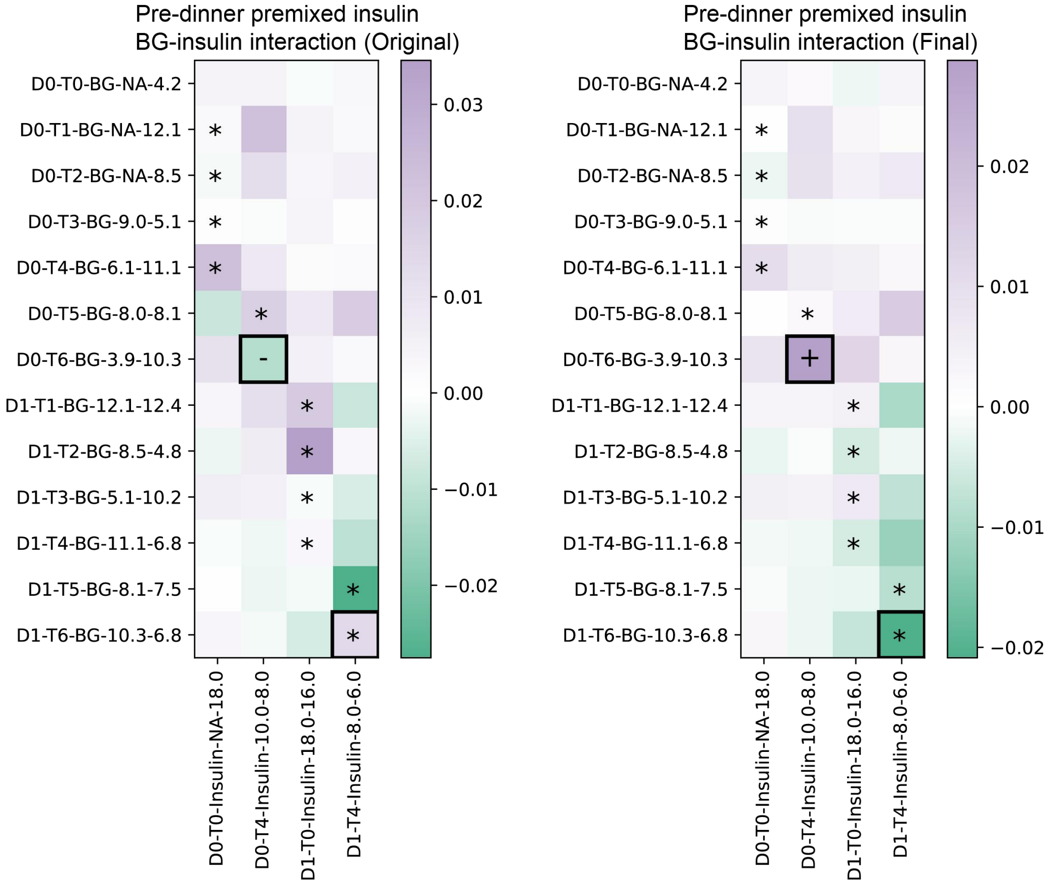

An example of explanation of pre-dinner premixed insulin prediction. This patient's pre-dinner insulin dose was reduced from 10u to 8u the day before, but the post-lunch blood glucose dose the day before was increased from 3.9 mmol/L to 10.3 mmol/L. According to medical knowledge, this BG-insulin interaction should not have a negative effect value on prediction. The original version of the algorithm assumes that the effect value is negative, which is wrong (Left). After adding the constraint, the final version of the algorithm considers this effect to be positive (Right).

Supplementary Figure 14. Expert evaluation results of alignment between the original and final version.

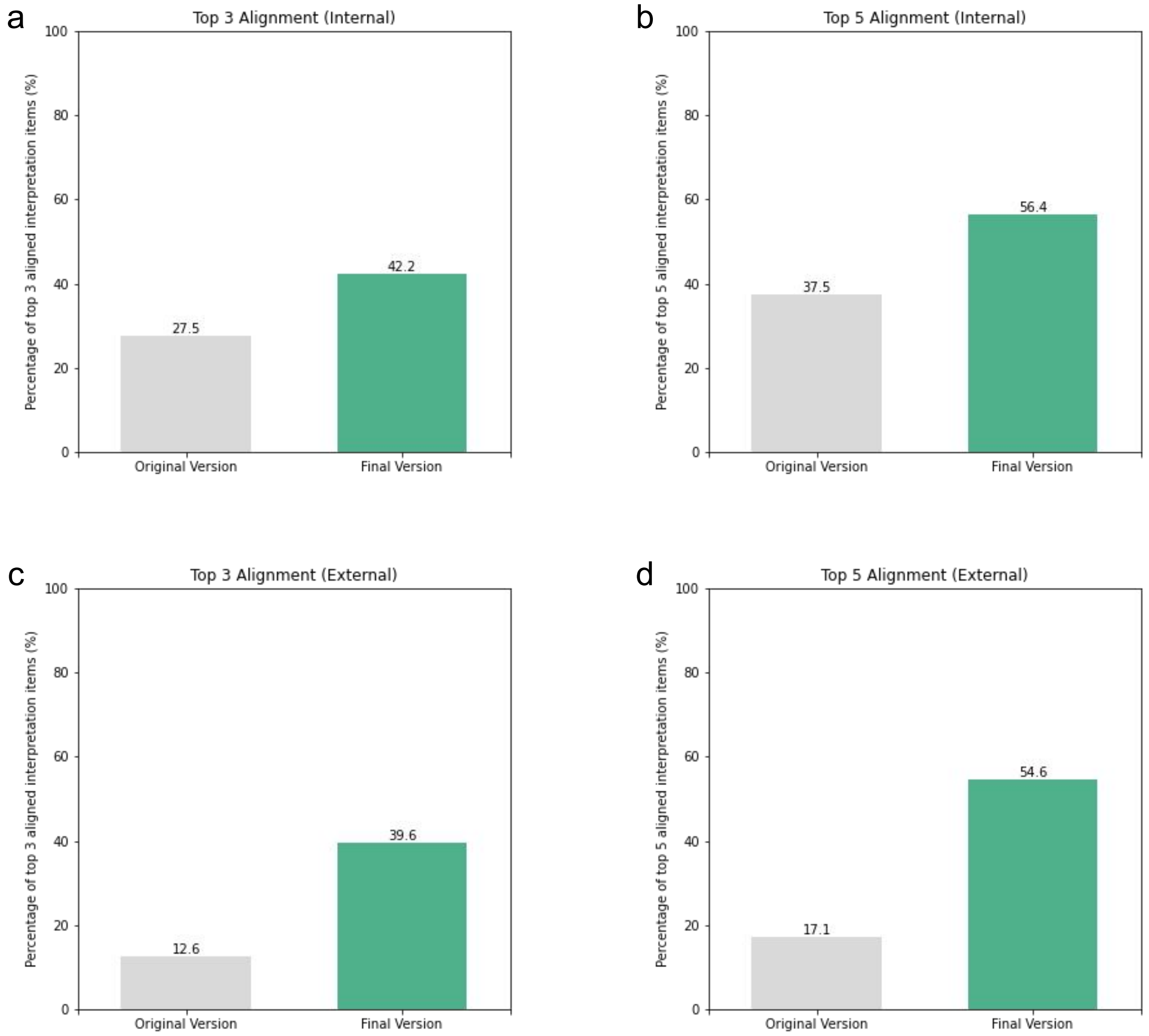

Comparison of original and final explanation versions on (**a-b**) an internal dataset (n = 20 regimens, 40 insulin points) and (**c-d**) an external dataset (n = 20 regimens, 46 insulin points) **(a, c)** Top 3 alignment rate. **(b, d)** Top 5 alignment rate.

Supplementary Figure 15. Comparisons of decision accuracy and confidence between junior and senior clinicians.

a

b

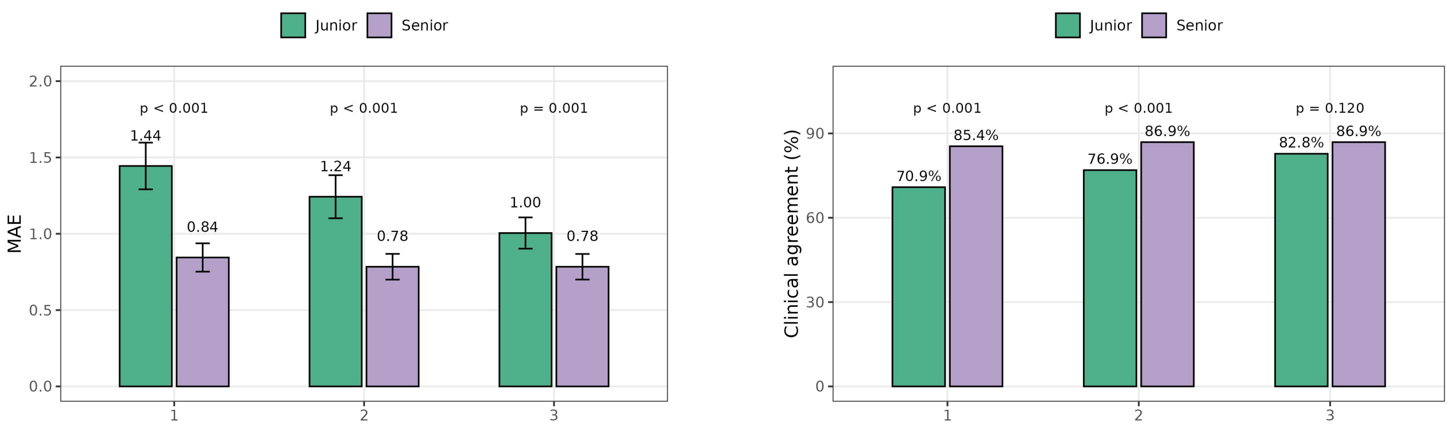

c

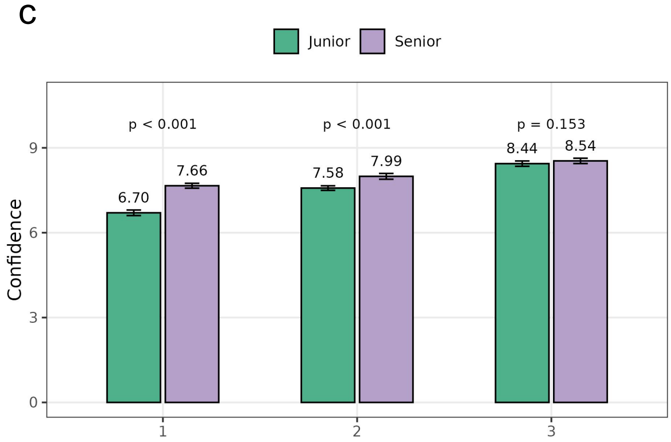

d

Outcome	Main effect of group	Main effect of scenario	Interaction (group × scenario)
MAE	F(1,6) = 16.55 p = 0.0066	F(2,2460) = 9.51 p < 0.001	F(2,2460) = 5.57 p = 0.0039
Clinical agreement	$\chi^2(1) = 13.79$ p < 0.001	$\chi^2(2) = 6.67$ p = 0.035	NS (p = 0.27)
Confidence	F(1,6) = 38.96 p < 0.001	$\chi^2(2) = 6.67$ p = 0.035	F(2,2460) = 42.22 p < 0.001

(a-c) Comparison of **(a)** Mean Absolute Error (MAE), **(b)** clinical agreement, and **(c)** clinician confidence across three scenarios: Scenario 1 (no AI assistance), Scenario 2 (plain AI dosage assistance), and Scenario 3 (explainable DIL system assistance). P-values were calculated using a t-test. **(d)** Linear mixed-effects models were used for MAE and confidence, while a generalized linear mixed-effects model with a logit link was used for clinical agreement. The models included fixed effects for clinician group, scenario, and their interaction (group × scenario), as well as random intercepts for clinician and case ID (for clinical agreement). P-values were obtained using Type III ANOVA with Satterthwaite's method; non-significant (NS) results are indicated when $p \geq 0.05$.

Supplementary Table 1. Characteristics of datasets in human evaluations.

	Internal evaluation set	External evaluation set	Clinical assistance set
Participants	20	20	40
Insulin counts/ Interpretation items	400	460	1030
Age (year)	66 (11)	75(12)	61 (13)
Sex			
Male (%)	9 (45%)	10 (50%)	19 (47.5%)
Female (%)	11 (55%)	10 (50%)	21 (52.5%)
Weight (kg)	66.0 (10.0)	67.6 (7.1)	68.5 (19.6)
BMI (kg/m^2)	25.8 (2.9)	24.2(1.8)	24.6 (4.7)
HbA1c (%)	9.3 (1.9)	8.4 (1.6)	9.2 (2.2)

Data are n (%) or mean (SD). BMI, Body Mass Index; HbA1c, glycated hemoglobin.

Supplementary Note 1. Questionnaire 1: Doctor in the loop

Task: Please VIEW the case, then carefully READ the explanation items of each recommended insulin point. You need to JUDGE if the item is appropriate or not based on guideline consensus and your personal clinical expertise.
If inappropriate, please indicate which of the three components resulted in an inappropriate item.

1) If the feature name is wrong, that is, you believe that the item does not affect the insulin dose, please mark the feature name.
2) If the effect direction is wrong, that is, you believe that the effect direction of item is not align with medical knowledge, please mark it and describe how the direction of item should be.
3) If the effect size is wrong, that is, you believe that the effect size of item is not suitable, please mark it and describe what the suitable effect size should be.
4) If there are other inappropriate items and suggestions, please provide.

Case ID______
Recommended Insulin Point
Insulin type______(basal/premix/shot)
Injection time______ (pre-breakfast; pre-lunch; pre-dinner; before-bedtime)

Item	Appropriate (Y/N)	Type			Suggestions for improvement
		Feature	Direction	Effect size	1.For feature, describe which feature is not relevant to insulin dose adjustment.<br>2.For direction, describe how the direction of item should be.<br>3.For effect size, describe what the suitable effect size should be.
1		☐	☐	☐	
2		☐	☐	☐	
3		☐	☐	☐	
4		☐	☐	☐	
5		☐	☐	☐	
6		☐	☐	☐	
7		☐	☐	☐	
8		☐	☐	☐	
9		☐	☐	☐	
10		☐	☐	☐	

Others ____

Supplementary Note 2. Questionnaire 2: Expert evaluation

Task: Please VIEW the case, then carefully READ the explanation items of each recommended insulin point. You need to GIVE the top 3 important feature of the insulin dosage adjustment based on medical consensus and your clinical experience. After that, please EVALUATE correctness.

Case ID______

Recommended Insulin Point

Insulin type______(basal/premix/shot)

Injection time______ (pre-breakfast; pre-lunch; pre-dinner; before-bedtime)

1. Please write the top 3 features

____

____

____

2. Please assess correctness (including feature, effect direction and effect size) for each item.

Item	Correctness		
	Feature (Y/N)	Direction (Y/N)	Effect size (Y/N)
1	☐	☐	☐
2	☐	☐	☐
3	☐	☐	☐
4	☐	☐	☐
5	☐	☐	☐
6	☐	☐	☐
7	☐	☐	☐
8	☐	☐	☐
9	☐	☐	☐
10	☐	☐	☐

Supplementary Note 3. Questionnaire 3: AI-assistance study

Task: Please VIEW the case, then carefully READ the explanation items of each recommended insulin point. You need to give your recommendation insulin dosage and score your decision confidence（1-10）.

Case ID______

Recommended Insulin Point

Insulin type______(basal/premix/shot)

Injection time______ (pre-breakfast; pre-lunch; pre-dinner; before-bedtime)

Step 1： Recommended dose and confidence with no AI assistance

Your recommended insulin dosage: _______ u

Confidence: _______ (1-10)

Step 2： Recommended dose and confidence with AI dosage assistance

Your recommended insulin dosage: _______ u

Confidence: _______ (1-10)

Step 3： Recommended dose and confidence with explanation AI assistance

Your recommended insulin dosage: _______ u

Confidence: _______ (1-10)
