# Supplementary material for: Explainable deep learning framework incorporating medical knowledge for insulin titration in diabetes
Source: Commun Med (Lond). 2026 Feb 26;6:192. doi: 10.1038/s43856-026-01449-1 (PMC13062103; doi:10.1038/s43856-026-01449-1)
Supplement: Supplementary file 2 — Description of Additional Supplementary files [file 43856_2026_1449_MOESM2_ESM.pdf]

Description of Additional Supplementary Files

Supplementary Data 1: The source data for Figures 2a-f, 2g, 2i, 3b-f, 4, and 6

Supplementary Data 2: Source data for Figure 2h

Supplementary Data 3: Detailed information of constraints
