# Supplementary material for: Explainable deep learning framework incorporating medical knowledge for insulin titration in diabetes
Source: Commun Med (Lond). 2026 Feb 26;6:192. doi: 10.1038/s43856-026-01449-1 (PMC13062103; doi:10.1038/s43856-026-01449-1)
Supplement: Supplementary file 5 — Supplementary data3 [file 43856_2026_1449_MOESM5_ESM.docx]

**Supplementary Data 3. Detailed information of constraints**

Here we introduce the constraints added in the final version with the assistance of endocrinologists. The constraints include general positive and negative constraints, prescribed constraints on glucose dimensions, missing dimension constraints, blood glucose value ratio constraints, and soft percentage constraints. We assume that "vector" refers to a column vector in the following context. We denote the input of insulin prediction network as $x$, and $x_{i}$ represents the $i$-th dimension of $x$. Note that $x=x^{T}-x^{T-1}$, where $x^{T}$ is today’s data and $x^{T-1}$ is yesterday’s data (that is the baseline data used to compare for Shapley series algorithms).

Notation:

$D_{pos}$ and $D_{neg}$ are positive and negative dimension sets summarized by experts, and $ReLU\left( x \right)=max(x, 0)$.

$D_{inc-sugar-insulin-inter}$ represents the glucose-insulin interaction dimensions with increasing glucose records, which is determined by each patient’s unique status.

$D_{dec-glucose-insulin-inter}$ represents the glucose-insulin interaction dimensions with decreasing glucose records

$D_{single-pos}$ and $D_{single-neg}$ are single positive and negative dimensions, and then $i,j$ determines the interaction dimensions of $\alpha$.

$D_{prescribed-glucose}$ is the insulin type-sensitive (e.g., long-acting, premixed or short-acting) time-dependent glucose dimensions determined by the guides and expert feedback.

$D_{missing}$ is the missing dimension set determined by each patient’s unique status.

$M_{glucose}\in\left\{ 0, 1 \right\}^{d^{2}+d}$ is the masking vector. We denote $m_{glucose}\in\left\{ 0, 1 \right\}^{d}$ as the masking vector for glucose dimensions. Then $M_{glucose}=Concate(m_{glucose} , m_{glucose}\bullet\vec{1}^{T} \wedge\vec{1} \bullet{m_{glucose}}^{T})$ , where $T$ denotes transpose, $\bullet$ denotes vector product, $\wedge$ denotes the logic and operation, and $Concate$ represents the concatenation operation between vectors and matrices. Similarly, $x_{sec}=Concate(x , \frac{x\bullet\vec{1}^{T}+\vec{1} \bullet x^{T}}{2})$. Also, $<,>$ is the inner product operation, and $\odot$ is the point multiply operation.

$\alpha_{\left[ 1:d \right]}$ represents the first $d$ dimensions of $\alpha$ corresponding to single dimensions.

| Constrains | Formula | Notation | Examples |
| --- | --- | --- | --- |
| BG change | $\sum_{i\in D_{pos}} ReLU({-\alpha}_{i})$ |  | The effect size of “increasing blood glucose” should be positive. |
|  | $\sum_{i\in D_{neg}} ReLU(\alpha_{i})$ |  | The effect size of “decreasing blood glucose” should be negative. |
| Antidiabetics medications | $\sum_{i\in D_{pos}} ReLU({-\alpha}_{i})$ |  | The effect size of “removing antidiabetic medications” should be positive. |
|  | $\sum_{i\in D_{neg}} ReLU(\alpha_{i})$ |  | The effect size of “adding antidiabetic medications” should be negative. |
| Prescribed BG | $ReLU(\lambda-\sqrt{\sum_{i\in D_{prescribed-glucose}} \frac{\alpha_{i}^{2}}{\left\vert\left\vert\alpha\right\vert\right\vert_{2}}})$ | On account of the importance of guideline-based glucose items, we made the percentage of these prescribed glucose items account for at least λ (0.25). was determined by. Notice that encouraging the effect size of prescribed glucose is equivalent to restricting non-prescribed glucose. And based on pharmacokinetics, different types of insulin should have different action times and therefore different ranges of prescribed glucose | Prebreakfast long-acting insulin injection requires more attention to BG throughout the day, and injection before bedtime requires more attention to BG before breakfast the next day. |
|  |  |  | Premix insulin injection should pay more attention to the pre-meal BG of the current meal and the next meal. |
|  |  |  | Short-acting insulin should pay more attention to the BG after the meal. |
| Missing content | $\sqrt{\sum_{i\in D_{missing}} \frac{\alpha_{i}^{2}}{\left\vert\left\vert\alpha\right\vert\right\vert_{2}}}$ | We would like the items included missing values had little influence on the insulin prediction. | The effect size of “None” (missing value) should be small. |
| BG-insulin Interaction | $\sum_{i\in D_{inc-glucose-insulin-inter}} ReLU\left( {-\alpha}_{i} \right)$  $if insulin is increased$ |  | BG was still increasing despite doctors had increased insulin dosage, under which conditions we would like the effect size to be positive. |
|  | $\sum_{i\in D_{inc-glucose-insulin-inter}} ReLU\left( {-\alpha}_{i} \right)$  $if insulin is decreased and x_{i}^{T} is hyperglycemia$ | Note that only prescribed BG record and insulin pairs are included. And hyper/hypoglycemia is time-dependent. For example, the standard BG range before meal is 3.9 to 6.1mmol/L, and BG range after meal is 6.1 to 8.0 mmol/L. | BG was increasing and above normal range and the doctors had decreased insulin dosage. Under this condition, we would like the effect size to be not negative (not decrease insulin dosage). |
|  | $\sum_{i\in D_{inc-glucose-insulin-inter}} ReLU\left( \alpha_{i} \right)$  $if insulin is decreased and x_{i}^{T} is hypoglycemia$ |  | BG was increasing but below normal range despite that the doctors had reduced insulin dosage. Under this condition, we would like the effect size to be negative. |
|  | $\sum_{i\in D_{dec-glucose-insulin-inter}} ReLU\left( {-\alpha}_{i} \right) if x_{i}^{T}is hyperglycemia$,  $\sum_{i\in D_{dec-glucose-insulin-inter}} ReLU(\alpha_{i}) if x_{i}^{T}is hypoglycemia$ |  | According to patient’s previous data, if BG is decreasing, we would like the positive or negative contribution be determined by the blood sugar status (hyperglycemia or hypoglycemia) of the day. |
